# Study of the Relationship Between the Structures and Biological Activity of Herbicides Derived from Phenoxyacetic Acid

**DOI:** 10.3390/ma18071680

**Published:** 2025-04-07

**Authors:** Grzegorz Świderski, Natalia Kowalczyk, Gabriela Tyniecka, Monika Kalinowska, Renata Łyszczek, Aleksandra Bocian, Ewa Ciszkowicz, Leszek Siergiejczyk, Małgorzata Pawłowska, Jacek Czerwiński

**Affiliations:** 1 Department of Chemistry, Biology and Biotechnology, Bialystok University of Technology, Wiejska 45E, 15-351 Białystok, Polandgabriela.tyniecka@sd.pb.edu.pl (G.T.);; 2 Department of Coordination and General Chemistry, Maria Curie Skłodowska University, M. Curie Skłodowska Sq. 2, 20-031 Lublin, Poland; renata.lyszczek@mail.umcs.pl; 3 Department of Biotechnology and Bioinformatics, Faculty of Chemistry, Rzeszow University of Technology, Powstańców Warszawy 6, 35-959 Rzeszow, Poland; bocian@prz.edu.pl (A.B.); ewa.ciszkowicz@prz.edu.pl (E.C.); 4 Institute of Chemistry, University of Białystok, K. Ciołkowskiego 1K, 15-245 Białystok, Poland; 5 Faculty of Environmental Engineering, Lublin University of Technology, Nadbystrzycka 40B, 20-618 Lublin, Poland; m.pawlowska@pollub.pl (M.P.); j.czerwinski@pollub.pl (J.C.)

**Keywords:** herbicides, MCPA, 2,4D, phenoxyacetic acid, antimicrobial study, cytotoxic study

## Abstract

Chloroderivatives of phenoxyacetic acid are a group of compounds commonly used as plant protection products. Differences in the molecular structure of these compounds are related to varying substitution and the number of chlorine atoms in the aromatic ring. Different molecular structures may affect the activity of these compounds, their physicochemical properties, as well as their toxicity and biological effects. A group of 6 chemical compounds derived from phenoxyacetic acid was tested. The molecular structure was analysed using spectroscopic methods (FTIR, FTRaman, UV-VIS, ^1^HNMR, ^13^CNMR) and quantum chemical computational methods (DFT). The reactivity of the tested compounds was determined using DFT calculations and experimentally in reaction with a hydroxyl radical. The electronic charge distribution of NBO, CHelpG and ESP was analysed and aromaticity indices were calculated for theoretically modeled structures and structures examined by X-ray diffraction (data obtained from the CSD database). Phenoxyacetic acid derivatives were tested for antimicrobial activity on soil bacterial strains. Cytotoxicity tests were performed on normal human skin fibroblasts (BJ CRL-2522) and the human prostate cancer cell line (DU-145 HTB-81). The purpose of this study was to investigate the relationship between the molecular structure of phenoxyacetic acid derivatives and their reactivity and biological activity.

## 1. Introduction

**Figure 1:**
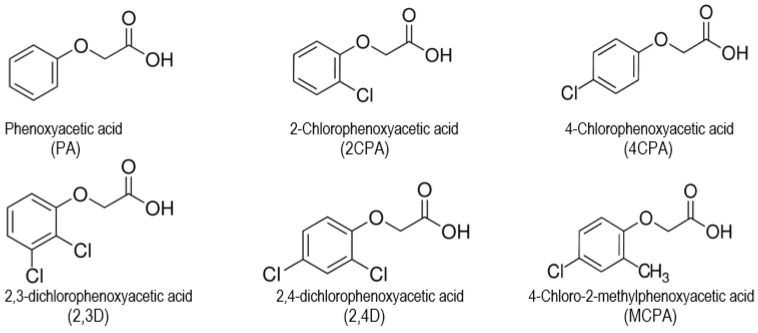
Molecular structure of phenoxyacetic acid and their chlorinated derivatives.

Chlorinated phenoxyacetic acid derivatives are a group of herbicides commonly used to control broadleaf weeds [1,2,3] (Figure 1). Phenoxy herbicides are introduced into the environment in various forms, i.e., free acids, alkali metal salts (usually sodium salt), diethylamines or esters. In the aqueous environment, these compounds dissociate and acidic anions are formed, which persist in the soil for about one month [4]. Herbicides used to protect plants enter the soil environment in significant amounts. Hence, they can migrate to groundwater and surface water, causing environmental pollution not only in the place of their application, but also in other areas where they were not originally used [5]. Active substances that are components of herbicides are toxic not only to humans and animal organisms, but they can also disrupt soil biocenosis [6]. For living organisms, not only the substances contained in herbicides are harmful, but also the products of their decomposition in the environment and metabolites formed by biochemical transformations [7]. Plant protection products can infiltrate into food and can increase the risk of cancer [8,9] and other diseases [10].

The data presented in numerous articles, literature reviews and WHO reports are unclear as to the genotoxic and carcinogenic effects of the most commonly used herbicides from the phenoxy acid group (MCPA, 2,4D) [11,12,13,14,15,16]. Laboratory tests on animals (rats and mice) did not show any carcinogenic effects of active substances used in herbicides [17,18,19]. However, epidemiological studies indicate a relationship between the risk of exposure to chlorophenoxy acid herbicides and the occurrence of cancer, especially soft-tissue sarcoma and non-Hodgkin lymphoma [16,20,21]. In this case, it is recommended to be very careful in interpreting the results [22,23].

Studies have shown that the active substances included in herbicides increase the level of oxidative stress in cells, which in the further stage affects the formation of inflammation and can initiate cancer processes [24,25,26]. Studies on the degree of lipid peroxidation by chlorinated phenoxyacetic acids (2,4D, 2,4,5T, MCPA) and their metabolites conducted by Duchniewicz and others showed that the highest degree of lipid peroxidation is caused by 2,4D. The introduction of a methyl substituent in place of chlorine, i.e., in the case of MCPA, causes the degree of peroxidation to be lower. An increase in the number of chlorine atoms in the aromatic ring of the herbicide also reduces the ability to peroxidise lipids. In the case of 2,4,5 T (3 chlorine atoms), the peroxidation capacity decreases compared to 2,4D (2 chlorine atoms) [7]. The effect of phenoxyacetate herbicides and their metabolites on eryrocytes was also examined. It was shown that at a concentration of 4 mM herbicide 2,4D caused hemolysis of 3.23% of erythrocytes, MCPA caused a slightly higher percentage—3.53% and 2,4,5T—6.62%. It was also observed that the metabolites of the tested herbicides (chlorophenols) showed a 3–4 times higher degree of erythrocyte damage than herbicides [7].

The biological activity, including the toxic properties of chemical compounds (including herbicides), is determined by their molecular structure, reactivity and ability to penetrate biological membranes of living organisms [27]. Herbicides from the group of chlorophenoxyacetic acids are chemical compounds that differ in terms of the number of substituents (chlorine, methyl group) and the place of their substitution in the aromatic ring. The biological activity, toxicity and weed control effectiveness by phenoxyacetic acid derivatives change along with the number and places of substitution of chlorine atoms in the molecule or the presence of a methyl substituent. The toxic properties of herbicides from the group of chlorophenoxy acids and their derivatives are influenced by environmental pH [28,29]. Cabral et al., in their toxicity tests of 2,4D and MCPA on Saccharomyces cerevisae, showed that at low pH, close to the pKa value of acids (2.73 and 3.07, respectively), toxicity is the highest and decreases with increasing pH. In the undissociated form of the acid, the toxicity is higher due to its increased ability to dissolve in fats [28,29].

The physicochemical properties and biological activity of chemical compounds are determined by their molecular structure and the distribution of electronic charge in molecules. By introducing chloro-substituents into the aromatic ring of phenoxyacetic acid the electronic structure of ligand and thus change their biological activity can be changed. The physicochemical properties, reactivity of the compound, its biological effects, including toxicity, will depend on the number of substituted chlorine atoms and the places of their substitution.

For example, the toxicity of chlorinated disubstituted anilines is correlated with the distance of the chlorine atoms from the amino group. However, there are also deviations from this relationship for some isomers, which is explained by a different mechanism of toxic action [30]. Many works on QSAR analysis have used DFT calculations, including reactivity descriptors based on the energy of HOMO and LUMO orbitals [31,32,33]

Padmanabhan et al. [34] analyzed the structure-reactivity relationship of 12 chlorinated benzene derivatives. The structure and reactivity of the studied compounds were investigated using DFT theory. Reactivity descriptors based on the energy of the frontal orbitals (HOMO and LUMO) were calculated. The results of the study showed that chlorinated benzene derivatives act as electron acceptors in reactions with biomolecules, and the electrophilicity index can act as an indicator of toxicity [34].

The study examined the physicochemical properties, molecular structure, electronic charge distribution and toxicity of chlorinated phenoxyacetic acid derivatives used as plant protection products. The research used spectroscopic methods (IR, Raman, NMR, UV electron absorption spectroscopy), molecular modeling using quantum chemical computational methods (reactivity descriptors—HOMO, LUMO and others) and biological tests. The relationship between the structure of the tested compounds and their biological activity was assessed.

## 2. Materials and Methods

### 2.1. Theoretical Studies

#### 2.1.1. Structures and Electronic Charge Distribution

The structure and infrared (IR) vibration frequencies of phenoxyacetic acid and its chlorine derivatives were calculated using the B3LYP/6-311++G(d,p) method [35]. The calculations took into account the possibility of the occurrence of conformational structures of acids and the results were compared with the structures solved by X-ray diffraction described in the literature. Theoretical wavenumbers were scaled according to the formula: ν_scaled_ = 0.98 × ν_calculated_ for B3LYP/6-311++G(d,p) level method. The chemical shifts were calculated using GIAO (gauge including atomic orbitals) method in the B3LYP/6-311++G(d,p) level. Theoretical values of chemical shifts were calculated by the GIAO method [36] in B3LYP/6-311+G(d,p) using DMSO as a solvent. Chemical shifts (δi) were calculated by subtracting the appropriate isotopic part of the shielding tensor (σi) from that of TMS (σTMS): δi = σ_TMS_ − σi (ppm). The isotropic shielding constants for TMS calculated by using the same basis set were equal to 31.8201 ppm for the 1H nuclei and 182.4485 ppm for the 13C nuclei, as calculated at the B3LYP levels, respectively. The electronic charge distribution of the studied molecules was calculated using the NBO [37] and CHelpG methods [38]. The electrostatic potential (ESP) distribution maps were calculated using the SCF for optimised structures calculated in the B3LYP/6-311++G(d,p) method.

The energy of HOMO and LUMO orbitals were calculated using the B3LYP/6-311+G(d,p) method. On the basis of the obtained HOMO and LUMO orbitals energy values, other reactivity descriptors, such as energy gap, ionization potential, electron affinity, electronegativity, chemical potential, hardness and softness, and electrophilicity index were calculated. Theoretical UV-VIS spectra were calculated for the modeled structures using the SDS solvent computational model in aqueous and methanol solution.

#### 2.1.2. Aromaticity

The aromaticity indices were calculated from the length of the bonds in the aromatic ring of the optimised structures. The values of aromaticity indices were calculated for theoretically modeled structures were compared with the values for structures developed using the X-ray diffraction method available in the CSD database. The following aromaticity indices were calculated based on the geometry of the aromatic ring of the tested compounds.

The Julg index (Aj) is calculated from the equation [39]:

$$A_{j}=1-\left(\frac{225}{n}\right){\sum_{r=1}^{n}\left\{1-\left(\frac{R_{r}}{R}\right)\right\}}^{2}$$

where n—number of CC bonds in the π-electron system; R_r_—current CC bond lengths; R—average value of the length of all CC bonds.

The BAC (Bond Alternation Coefficient) index is calculated from the equation [40]:

$$BAC=1-3.46\sqrt{\sum_{n}{\left(R_{n}-R_{n+1}\right)}^{2}}$$

where R_n_—length of the nth bond; R_n+1_– length of the bond adjacent to the nth.

The HOMA index (Harmonic Oscillator Model of Aromaticity) and its EN—energetic and GEO—geometric components are calculated from the formula [41]:

$$HOMA=1-\left[\alpha {\left(R_{opt}-R_{av}\right)}^{2}+\frac{\alpha }{n}{\sum \left(R_{av}-R_{i}\right)}^{2}\right]=1-EN-GEO$$

where R_av_—average bond length; n—number of bonds considered; R_opt_—optimal binding length; R_i_—length of the i-th bond; α—normalisation coefficient.

Bird’s index, is calculated from the equation [42]:

I = 100[1 − (V/V_k_)]

where Vk is 35 for five-membered rings, and six-membered ones 33.3.

V—can be calculated from the equation:

$$V=\left(100/n_{av}\right){\left[\sum_{r=1}^{n}{\left(n_{r}-n_{av}\right)}^{2}/n\right]}^{\frac{1}{2}}$$

where n_av_—average bond order, n—number of bonds, n_r_—bond order.

For purely aromatic systems (benzene), the values of the BAC, Aj and HOMA indices take the value 1, while for non-aromatic systems (cycloheksatriene) the value is 0. In the case of the Bird index, the value scale is from 0 to 100.

#### 2.1.3. Reactivity

**Figure 2:**
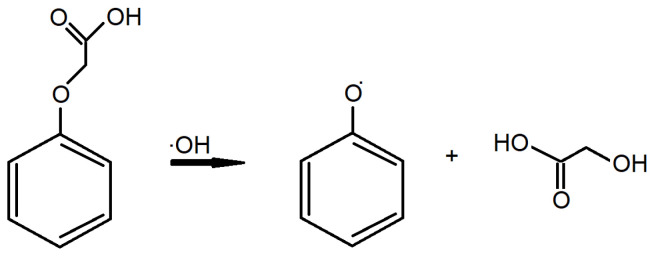
Reaction of hydroxyl radical with phenoxyacetic acid.

The reactivity of the tested compounds was tested based on the reaction with the hydroxyl radical. OH occurring according to the scheme shown in Figure 2 [43].

The energy of the presented reaction ΔE is:

ΔE = [E_(PR)_ + E_(GA)_] − [E_(PA)_ + E_(.OH)_]

where ΔE—reaction energy, E_(PR)_—Energy of the phenoxy radical, E_(GA)_—Energy of the glicolic acid, E_(PA)_—Energy of the phenoxyacetic acid, E_(.OH)_—Energy of the hydroxylic radical.

**Figure 3:**
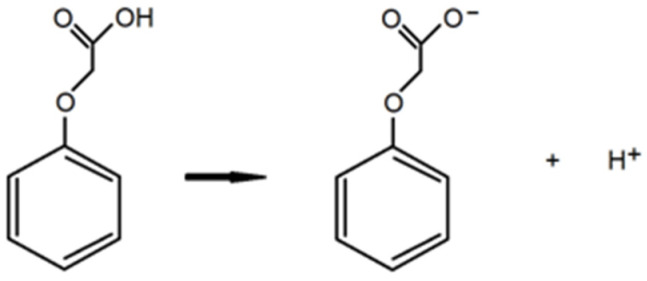
Reaction of deprotonation phenoxyacetic acid.

**Figure 4:**
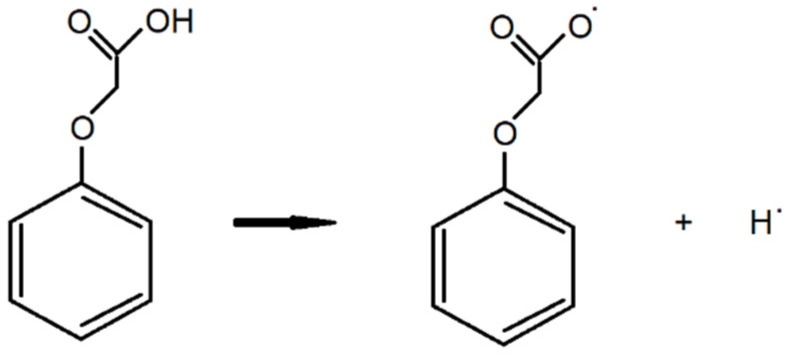
The reaction of the decomposition of phenoxyacetic acid into radicals.

The deprotonation energy DE and the BDE value (Bond Dissotiation Energy) of phenoxyacetic acids were also calculated based on the reactions shown in Figure 3 and Figure 4.

The deprotonation energy was calculated based on the equation:

DE = [E_(ion)_ + E_(H^+^)_] − E_(PA)_

where DE—deprotonation energy, E_(ion)_—energy of deprotoneted acid, E_(H^+^)_—proton energy, E_(PA)_—energy of protonated acid.

Bond dissociation energy (BDE) is a measure of the strength of the A-B chemical bond. It can be defined as the standard enthalpy change when A-B is cleaved by homolysis to produce fragments A and B, which are radicals. BDE (Bond dissotiation energy) is calculated from the equation below:

BDE = [H_(PA.)_ + H_(H.)_] − H_(PA)_

where H_(PA.)_—enthalpy of phenoxyacetic radical, H_(H.)_—enthalpy of hydrogen radical, H_(PA)_—enthalpy of phenoxyacetic acid.

The energy effects of the reaction were calculated using the DFT/B3LYP-6-311++G(d,p) method, taking into account the aqueous environment (CPCM computational model).

All calculations were performed using the Gaussian 09 software package [44].

### 2.2. HO^•^ Radical Inhibition Activity

Hydroxyl radical inhibition assay was performed according to [45]. 0.3 mL of FeSO_4_ (8 mM), 1 mL of salicylic acid ethanol solution (3 mM) and 0.25 mL of H_2_O_2_ (20 mM) were added to 1 mL of tested compound in the concentration range of 0.1 mM −1 µM. Control sample consisted the same amounts of compounds but H_2_O was used instead of H_2_O_2_. The blank sample consisted DMSO instead of tested compounds. All samples were incubated in 37 °C for 30 min, then 0.5 mL of H_2_O was added, and the absorbance was measured at λ = 510 nm. The inhibition level was calculated according to the formula:

$$I\%=\left(1-\left(\frac{A_{t}-A_{c}}{A_{b}}\right)\right)\cdot 100\%$$

where $A_{c}$—absorbance of the control sample, $A_{t}$—absorbance of the tested sample, $A_{b}$—absorbance of the blank sample.

### 2.3. Spectroscopic Studies

The IR spectra of derivatives from phenoxyacetic acid were performed using the method of pressing sample in KBr and ATR technique (Attenuated Total Reflectance) using an Alfa spectrometer (Bruker, Billerica, MA, USA). The spectra were recorded in the range of 400–4000 cm^−1^. The Raman spectra were recorded using a Multi Raman spectrophotometer (Bruker, Bremen, Germany) in the range of 400–4000 cm^−1^, with a laser power of 200 mW. The UV spectra of analywed compounds at the concentration of 5.10^−5^ mol/dm^3^ aqueos and methanol solution were recorded in the range of 190–400 nm using the UV/VIS/NIR Agilent Carry 5000 spectrophotometer (Santa Clara, CA, USA). The ^1^H (400.15 MHz) and ^13^C (100.63 MHz) spectra were registered with the Bruker Avance II 400 spectrometer (Bremen, Germany) in DMSO-D6 solution. TMS was used as an internal reference.

### 2.4. Antimicrobial Study

The in vitro antibacterial activity of herbicides was investigated against Gram-positive *Bacillus megaterium* (ATCC 14581) and Gram-negative *Pseudomonas aeruginosa* (ATCC 15442) after Karcz et al. 2022 [46] and according to the National Committee for Clinical Laboratory Standards guideline. The minimum inhibitory concentration (MIC) values were determined by visual and spectrophotometric measurement (λ = 600 nm, Varioskan™ LUX multimode microplate reader, Thermo Scientific^™^, Waltham, MA, USA) as the lowest concentration at which no bacterial growth is observed. The minimum bactericidal concentration (MBC) was determined by colony counting on Mueller-Hinton agar plates (MHA) as the lowest concentration that inhibits bacterial colony growth [47].

The initial 24-h bacterial culture was incubated at 37 °C in a New Brunswick Innova 40 Shaker (Eppendorf AG, Hamburg, Germany) and prepared by adjustment to the 0.5 McFarland standard (10^8^ colony forming units, CFU/mL). Serial dilution of herbicides was carried out to obtain concentrations from 3.05 µg/mL to 12.5 mg/ml. Bacterial cultures were diluted to a final density of 10^5^ CFU and added to each well of prepared plates with the exception of those used as medium sterility. After 24 h of incubation at 37 °C (atmospheric conditions), visual turbidity was observed, as well as optical density measurement at 600 nm using a Varioskan LUX^TM^ multimode microplate reader (Thermo Scientific™, Waltham, MA, USA). The medium without antibacterial agents and wells without bacterial cultures added served as a control of bacterial growth and a solvent control, respectively. An evaluation of the susceptibility of each bacterial strain to the gentamicin (GEN) and rifampicin (RIF) antibiotics was also performed using the microdilution method (concentration range from 1.91·10^−3^ to 500 µg/mL). All reagents and bacterial cultures were prepared under sterile conditions using ESCO Airstream Laminar Flow Cabinet (Esco Lifesciences GmbH, Friedberg, Germany). All experiments were carried out in triplicate to ensure the reproducibility of the results.

### 2.5. Cytotoxic Study

#### 2.5.1. Cell Culture and Herbicides Treatment

Normal human skin fibroblasts (BJ CRL-2522) and the human prostate cancer cell line (DU-145 HTB-81) were obtained from the American Type Culture Collection (ATCC) and maintained in the recommended cell culture medium and conditions of the American Type Culture Collection. Both cell lines were cultured in complete culture medium, composed of Eagle’s Minimum Essential Medium (EMEM ATCC 30-2003) supplemented with 10% fetal bovine serum (FBS ATCC 30-2020) in a humidified atmosphere containing 5% CO_2_ at 37 °C. The herbicides were dissolved in dimethyl sulfoxide (DMSO) and diluted to the required concentration with medium when needed. Before herbicide treatment, the cells were grown to a density of approximately 85–90% and then treated with herbicides at different concentrations for 24 h. Control cells were incubated with DMSO at a final concentration 0.4%.

#### 2.5.2. Cell Viability Assay

Cell viability was determined in vitro using the WST-1 assay (ab155902, Abcam, Inc., Cambridge, UK) according to the manufacturer’s instructions and after Miłek et al. (2022) [48]. Briefly, BJ and DU-145 cells were seeded in 96-well plates (10^4^ cells/well) and incubated overnight at 37 °C and 5% CO_2_. Cells were subjected to 24-h treatment with herbicide solutions at final concentrations of 25, 100 and 400 µg/mL. The cells that were incubated with an equivalent amount of DMSO without tested herbicides served as controls. Then, 10 µL of WST-1 reagent was added to each well and incubated for 2 h under the culture conditions described above. Spectrophotometric measurement was performed with a Varioskan LUX™ multimode microplate reader (Thermo Scientific™, Waltham, MA, USA) at a wavelength of 490 nm versus 630 nm to eliminate background factors. Cell viability was calculated and expressed as a percentage of control cells (nonexposed cells) [48]. The treatments were carried out in triplicate in two independent experiments.

#### 2.5.3. Statistical Analysis

Statistical analyses have been performed using GraphPad Prism 8 software (GraphPad Software Inc., San Diego, CA, USA). All data are expressed as means and standard deviations. Significant differences with *p* < 0.05 between control and treated cells at different concentrations of herbicides were determined using the Kruskal-Wallis test.

## 3. Results and Discussion

### 3.1. Theoretical Calculations

#### 3.1.1. Structure and Aromaticity

**Figure 5:**
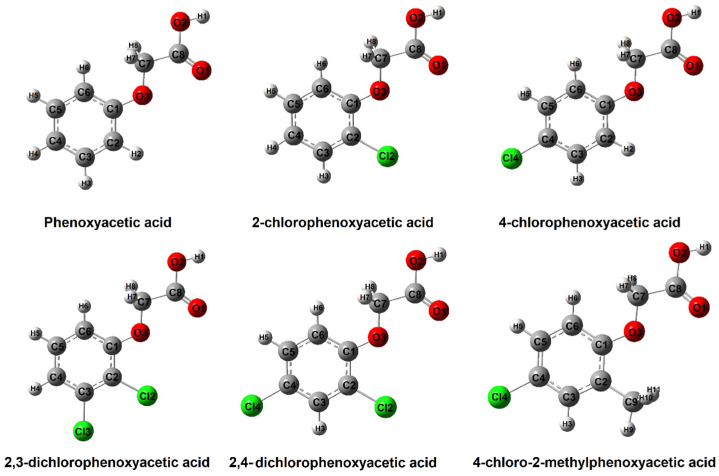
Monomer structures for phenoxyacetic acid (optimal geometry conformers) and chlorinated derivatives calculated in B3LYP/6-311++G(d,p).

X-Ray data indicate that phenoxyacetic acid and its chlorosubstituted derivatives adopt a (syn-syn) conformation. Theoretical calculations of the conformational structures of the tested compounds showed that this type of conformation is energetically favored. The theoretically modeled structures of phenoxyacetic acid and its chlorosubstituted derivatives are presented in Figure 5.

Table 1 lists the geometric and energetic parameters of the modeled structures of phenoxyacetic acid and its chlorine derivatives. On the basis of the bond lengths in the aromatic ring of the tested compounds, aromaticity indices were calculated, which are a quantitative measure of the delocalisation of the π-electron system. Geometric aromatic indices allow determining aromaticity based on the degree of variation in the length of bonds in the ring.

The location/delocalisation of electrons is crucial in the stability of the molecule and its reactivity (susceptibility of the aromatic ring to substitution). Unsubstituted phenoxyacetic acid has the highest aromaticity among the tested compounds. The influence of substituents on π-electron systems characterised by high aromaticity is weaker than on systems with lower aromaticity. It was observed that the substitution of chlorine in the aromatic ring of phenoxyacetic acid, characterised by high aromaticity (HOMA = 0.983; I6 = 96.16), destabilises the π-electron system—an increase in bond alternation, which translates into a decrease in the value of the calculated aromaticity indices. However, this effect is negligible. The extent of destabilisation of the system by substituents depends on the place of substitution of chlorine in the aromatic ring in relation to the carboxyl group. Substituting the chlorine atom in position 4 (para) has a smaller impact on the destabilisation of the π-electron system than in the ortho and meta positions. Phenoxyacetic acid and its para-substituted chlorine derivatives are characterised by higher aromaticity than other molecules.

The total dipole moment reflects the ability of the tested molecule to interact with the surrounding environment (molecule polarity) [49]. The calculated values of the dipole moment of herbicide molecules showed that phenoxyacetic acid has the lowest polarity, while 2,3-dichlorophenoxyacetic acid has the highest polarity among the analysed herbicides. The change in polarity of the acids tested varies in the following series: 2,3D→2,4D→2CPA→4CPA→MCPA→PA.

#### 3.1.2. HOMO-LUMO Orbitals

The most important orbitals in a molecule are the frontier molecular orbitals (Highest Occupied Molecular Orbitals—HOMOs, and Lowest Unoccupied Molecular Orbitals—LUMOs). These orbitals determine the way the molecule interacts with other species. The frontier orbital gap helps to characterise the chemical reactivity and kinetic stability of the molecule [50,51].

The soft systems have small HOMO–LUMO gap, and highly polarisable. A large HOMO–LUMO gap implies high molecular stability and aromaticity low reactivity in chemical reactions [52,53] while a small HOMO–LUMO gap is related to anti-aromaticity. The HOMO and LUMO energies represent the ability to donate and gain an electron, respectively. The high HOMO energy corresponds to the more reactive molecule in the reactions with electrophiles while low LUMO energy for molecular reactions with nucleophiles [54]. On the basis of the energy values of the HOMO and LUMO orbitals, descriptors related to the reactivity of the molecules were calculated [55,56].

The ionisation potential is equivalent to the negative energy value of the HOMO orbital, i.e., IP = −E_HOMO_, while the electron affinity is the negative energy value of the LUMO orbital, i.e., EA = −E_LUMO_. Global reactivity descriptors are described by the equations presented below.

The chemical potential $\mu =-\frac{\left(IP+EA\right)}{2}$ expresses the electrons’ ability to detach and escape from a stable system. Chemical hardness $\eta =\frac{\left(IP-EA\right)}{2}$ determines the resistance to deformation of the electron cloud of a molecule under the influence of disturbances occurring in chemical reactions. Molecules with higher hardness are less susceptible to changes in the electronic charge distribution caused by the attachment of substituents, e.g., to the aromatic ring. The larger the energy gap (HOMO-LUMO), the less reactive a given molecule is (the molecule is hard in local terms). The inverse of the hardness of a molecule is the softness described by the equation $S=\frac{1}{2\eta }$.

Another descriptor is the electrophilicity index. Electrophilicity is related to the ability of an electrophilic molecule to acquire additional charge while resisting the exchange of electron charge with its surroundings. This property is related to the chemical potential (the ability to transfer charge) and the hardness of the molecule (its electronic stability). The electrophilicity index is described by the equation ω= μ^2^/2η, and provides information about the toxicity of molecules in the context of their reactivity [57].

**Figure 6:**
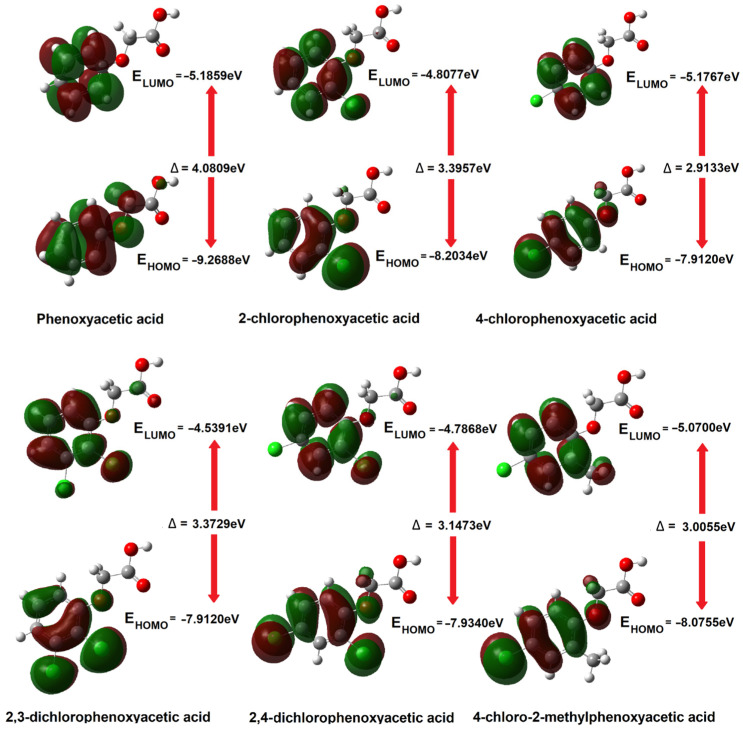
HOMO and LUMO orbitals contours calculated in B3LYP/6-311++G(d,p) (in vaccum) for phenoxyacetic acid and their chlorinated derivatives.

The HOMO and LUMO energy values and the reactivity descriptors calculated using the DFT method (B3LYP/6-311++G(d,p) are listed in Table 1. Figure 6 shows the shapes of the HOMO and LUMO orbitals for phenoxyacetic acid and its chlorine derivatives.

The highest GAP energy value was observed for phenoxyacetic acid, which is characterised by the lowest reactivity among the tested compounds. This compound has the highest hardness, which proves that it is characterised by the highest electronic charge stabilisation. Phenoxyacetic acid should therefore have the highest aromaticity among the tested compounds. Most of the calculated aromaticity indices actually indicate these relationships (except the HOMA index). According to the calculated GAP energy value, 4-chlorophenoxyacetic acid has the highest reactivity and the lowest hardness. However, this compound has the highest aromaticity, which would indicate that it is a compound with high electronic system stabilisation. The geometric criteria of aromaticity (differentiation of bond lengths in the aromatic ring) do not always reflect the stabilisation of the electronic system determined on the basis of energy data (obtained energy values of frontal orbitals). Substituting the chlorine atom in the aromatic ring of phenoxyacetic acid in position 2 increases the reactivity of the molecule and reduces its hardness. The aromaticity of the molecule also decreases. Substituting another chlorine atom in the aromatic ring causes a further increase in reactivity and a decrease in the hardness of the molecule, as well as a decrease in the aromaticity of the molecules. The reactivity and hardness of disubstituted derivatives of phenoxyacetic acid in positions 2 and 4, i.e., 2,4D and MCPA, are at a similar level, as is the aromaticity of both compounds. The increase in the reactivity of the tested compounds, in accordance with the change in GAP energy, can be arranged in the following series: PA→2CPA→2,3D→2,4D→MCPA→4CPA. In the same series, the hardness of the particles decreases. However, the decrease in aromaticity of the tested molecules based on the calculated geometric aromaticity indices can be summarised as follows: PA→4CPA→2CPA→2,4D→2,3D→MCPA (this is indicated by most of the calculated indices apart from the HOMA values). In general, it can be stated that the reactivity and electronic stability of chlorophenoxy acids are consistent with their aromaticity index values.

#### 3.1.3. Electronic Charge Distribution

**Figure 7:**
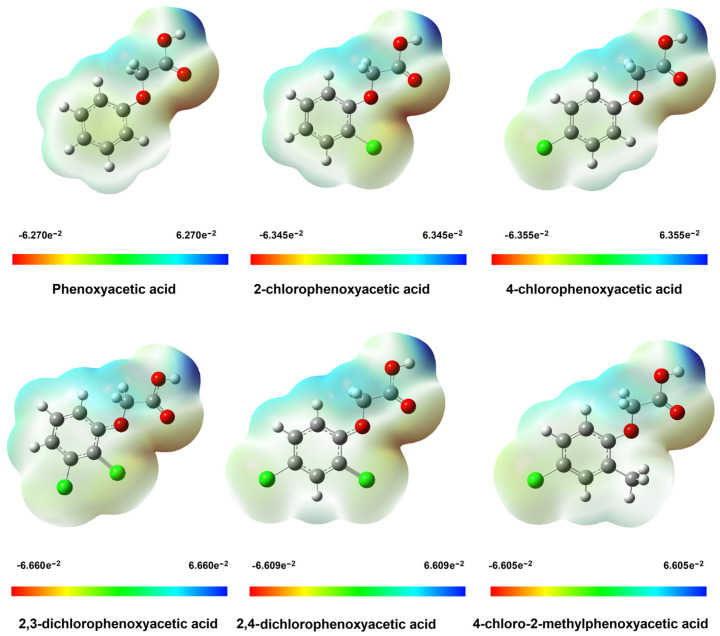
Electrostatic potential maps for phenoxyacetic acid and their chlorinated derivatives calculated in B3LYP/6-311++G(d,p).

The electrostatic potential map shows areas of electrophilic (red) and nucleophilic (blue) in molecules (Figure 7). In the phenoxyacetic acid molecule, increased susceptibility to electrophilic reactivity is characterised by the oxygen atom of the electrophilic group and, to a lesser extent, the oxygen atom connected directly to the aromatic ring. The nucleophilic region includes the oxygen atom of the hydroxyl group and the hydrogen atoms of the aliphatic carbon CH_2_. In the tested molecules, hydrogen atoms in the aromatic ring are susceptible to nucleophilic substitution. The protons of these groups are electron-poor due to the shift of the electron cloud towards electronegative oxygen atoms. The area encompassing the carbon atoms of the aromatic ring of phenoxyacetic acid is electrophilic. Substituting chlorine atoms, which are characterised by high electronegativity, causes the electron cloud to shift towards these atoms. The area encompassing the carbon atoms of the aromatic ring becomes nucleophilic. Areas of electrophilic nature in chlorine derivatives of phenoxyacetic acid are located around chlorine atoms. It can be seen that the reactivity of phenoxyacetic acid bound to the aromatic ring will vary with the site of substitution of the chlorine atom. This effect on the hydroxyl group located in the carboxyl group may be smaller.

##### NBO and CHelpG

The distribution of electronic charge on atoms in herbicide molecules was calculated in DFT/B3LYP/6-311++G(d,p) using two methods: NBO (Natural Bond Orbital) and CHelpG (Table S1). In the NBO method, bond orbitals with maximum electron density are calculated [37]. CHELPG (CHarges from ELectrostatic Potentials using a Grid-based method) is an atomic charge calculation scheme developed by Breneman and Wiberg in which atomic charges are adjusted to reproduce the molecular electrostatic potential (MESP) at multiple points around the molecule [38]. Charge distributions (shown in Table S1) were calculated for the modeled structures of herbicides in the gas phase and in water using the CPMC model. The differences in the values of the calculated charges in the gas phase and in the solvent—water were insignificant.

Negative charges are accumulated on the carbon atoms of the aromatic ring of phenoxyacetic acid, except for the carbon atom connected to the oxygen atom. The electronegative oxygen atom attracts the charge from the carbon atom that has a positive charge. Substitution of the chlorine atom (type I substituent) deactivates the ring and directs the substituents in the electrophilic substitution reaction in the ortho and para positions. It can be observed that the electron charge on the carbon atom to which chlorine is substituted decreases, while the electron charge on the carbon atom ortho relative to the site of substitution increases (in 2- and 4-chlorophenoxyacetic acids). Chlorine substitution causes destabilisation of the π-electron charge in monosubstituted derivatives of phenoxyacetic acid, and this effect is greater for substitution in the 2-position than in the 4-position. Substituting another chlorine atom in the aromatic ring causes an increase in the disturbance of the π-electron charge distribution. A stronger effect of shifting the electron cloud in the aromatic system towards electronegative substituents is observed. The effect of disturbing the electronic charge distribution is greater when the substituents are located in close proximity, as is the case in the 2,3-dichlorophenoxyacetic acid molecule.

In the case of 2,4-dichlorophenoxyacetic acid, the charge distribution in the aromatic ring will be more uniform (stable). As a result, 2,3D acid is a less stable (more reactive molecule) than 2,4D acid. The electronic charge distribution (NBO method) in the MCPA aromatic ring is similar to that in 2,4D acid. The electron density on the carbon atom connected to the methyl group in the MCPA molecule (in position 2) is slightly lower than the value for the carbon atom substituted with chlorine in position 2 in 2,4D acid.

The electronic stabilisation of the aromatic system described by the values of the calculated charge distribution using the NBO method in the tested series of herbicides can be presented as follows: PA→4CPA→2CPA→2,4D→MCPA→2,3D. These changes are identical to the change in aromaticity of the tested systems determined using geometric aromaticity indices. Calculations of the electronic charge distribution using the ChelpG method also showed that the substitution of the chlorine atom in the aromatic ring of phenoxyacetic acid causes destabilisation of the electronic system. The destabilising effect of the aromatic system depends on the position and number of electronegative substituents. Changes in the electronic charge distribution in the tested series of compounds calculated using the CHelpG method are the same as in the case of the charge distribution determined using the NBO method. The distribution of electronic charge in the carboxyl group and on the atoms of the aliphatic CH_2_ group is similar in all tested systems. No significant effect of substituting chlorine atoms in the aromatic ring of phenoxyacetic acid on the change in the values of NBO charges distributed outside the aromatic system was observed.

### 3.2. Results of Spectroscopic Studies

#### 3.2.1. FTIR and FTRaman

**Figure 8:**
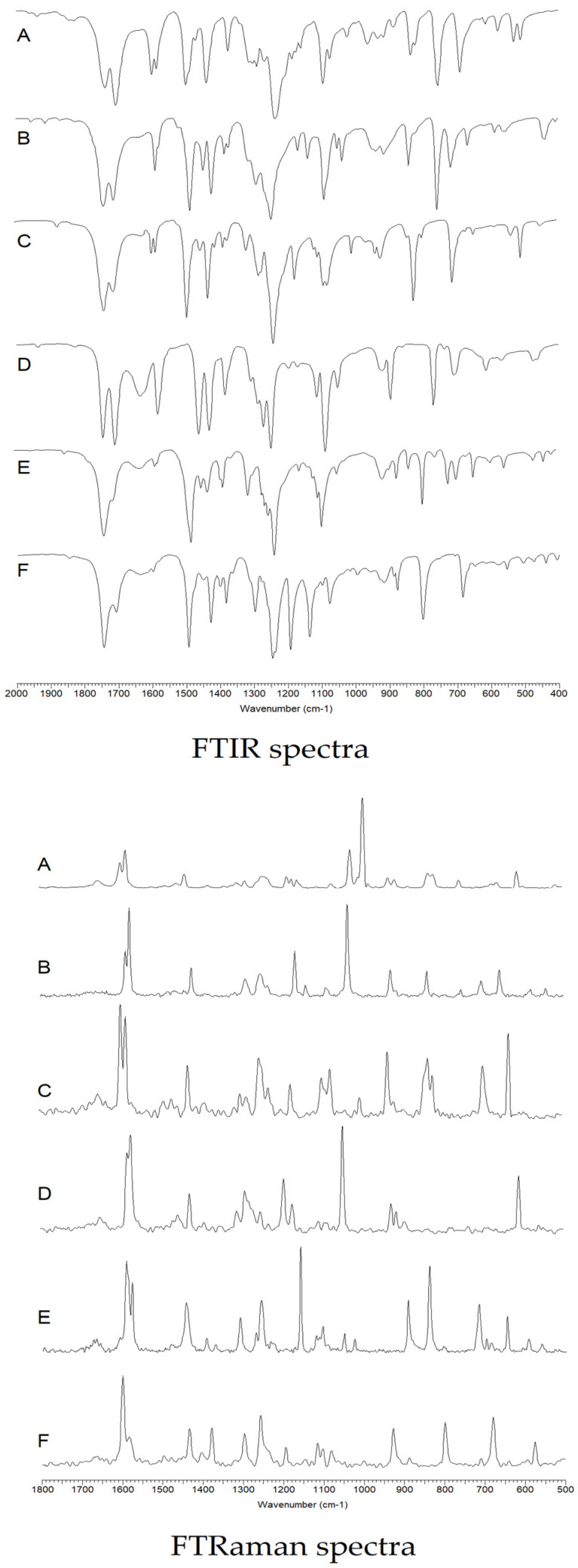
IR and Raman spectra registered in KBr. A phenoxyacetic acid, B 2-chlorophenoxyacetic acid, C 4-chlorophenoxyacetic acid, D 2,3-dichlorophenoxyacetic acid, E 2,4-dichlorophenoxyacetic acid, F 4-chloro-2-metylphenoxyacetic acid.

The infrared and Raman spectra recorded for chlorophenoxyacetic acid and its chlorine derivatives were assigned based on literature data and theoretical calculations of infrared spectra for modeled acid molecules using the density functional method (B3LYP/6-311++G(d,p). In the assignment of spectra, the notation according to Versanyi was used for the normal vibrations of the aromatic ring. The vibrations were visualised in the Gauss View program based on the calculated infrared vibrations for benzene using the B3LYP/6-311++G(d,p) method (Figure 8).

On the basis of the intensity and position of the bands of the aromatic system, changes in the electronic stability of the tested molecules can be assessed. The decrease in the intensity of the bands, their disappearance or shift towards lower wave numbers in the spectra of the compared compounds indicates a decrease in the stabilisation of the π-electronic system.

Figure 8 shows the FTIR infrared spectra recorded in the KBr matrix and the Raman spectra. Wavenumbers, FTIR band intensities (recorded in KBr and ATR), Raman, theoretically calculated band wavenumbers, and band assignments are summarised in Table 2 and Table 3.

On the basis of the obtained spectroscopic data, the change in the electronic charge distribution in the aromatic ring of the tested phenoxyacetic acids was analysed.

The infrared spectra of phenoxyacetic acid show stretching vibration bands of the carbonyl group νC = O present at wave numbers 1736 and 1703 cm^−1^ (IRKBr) and 1732 and 1702 cm^−1^ (IRATR). These bands are not observed in the Raman spectrum. The presence of two bands νC = O in the IR spectra indicates the formation of dimers by the acid. The band wavenumber value calculated using the DFT/B3LYP/6-311++G(d,p) method is 1814 cm^−1^. In the spectra of chlorinated derivatives of phenoxyacetic acid, a slight shift of the νC=O band towards higher wave numbers (up to approximately 1743 cm^−1^ and 1710 cm^−1^) is observed in comparision to the position of the band in the spectrum of the unsubstituted acid. Another high-intensity band present in the spectra of phenoxyacetic acids related to the vibration of the carboxyl group is the νC-OH stretching vibration band. In the spectra of phenoxyacetic acid, it is located at 1158 cm^−1^ (IRKBr), 1160 cm^−1^ (IRATR) and 1160 cm^−1^ (Raman). In the spectra of monochloro derivatives of phenoxyacetic acid, a decrease in the intensity of this band and a shift towards lower wave numbers (up to approximately 1137 cm^−1^) is observed. However, in the spectra of disubstituted chlorine derivatives, this band is not observed (in the spectrum of 2,3- and 2,4-dichlorophenoxyacetic acid). The bending vibration band of the βOH hydroxyl group is located on the spectrum of phenoxyacetic acid at values of 1300 cm^−1^ (IRKBr), 1307 cm^−1^ (IRATR) and 1306 cm^−1^ (Raman). The presence of this band was not observed in the spectra of chlorinated phenoxyacetic acid derivatives. It probably overlaps closely located vibrational bands of the aromatic system. The stretching vibration bands of asymmetric aliphatic carbons ν_as_CH_2_ in the spectrum of phenoxyacetic acid are located at wave numbers 2921 cm^−1^ (IRKBr), 2920 cm^−1^ (IRATR) and 2923 cm^−1^ (Raman). These bands shift on the spectra of chlorine derivatives are towards lower values in 2CPA, 2,3D and 2,4D and in 4CPA and MCPA towards higher wave number values. The stretching vibration bands of symmetric aliphatic carbons νsCH_2_ in the spectrum of phenoxyacetic acid are located at wave numbers 2799 cm^−1^ (IRKBr) and 2804 cm^−1^ (IRATR). These bands shift on the spectra of derivatives in 2CPA, 4CPA, 2,3D and 2,4D towards lower values. This band was not observed on the spectrum of MCPA.

Theoretical calculations of charge distribution and aromaticity showed that substituting chlorine atoms in the aromatic ring of phenoxyacetic acid increases the destabilisation of the electronic system. Analogous conclusions can be drawn based on the analysis of changes in the intensity of the bands and changes in the positions of the bands related to the vibrations of the aromatic system in the tested series of chlorinated derivatives of phenoxyacetic acid. A decrease in wave number values and the disappearance of the aromatic system bands numbered: 20a, 8b, 14, 12, 5, 10b, 10a, 6b, 16b were observed. The wavenumbers values of some bands present in the spectra of derivatives increase relative to the bands of the aromatic system of phenoxyacetic acid. These are the bands: 3, 9a, 9b, 17b, 11. On the basis of the analysis of band shifts in the spectra of the tested compounds, it was observed that the substitution of two chlorine atoms causes greater destabilisation of the aromatic system than the substitution of one chlorine atom. In the spectra of 2,3D and 2,4D, the disappearance of many bands of the aromatic system, which were present in the spectrum of phenoxyacetic acid, is observed. The analysis of the infrared and Raman spectra indicates that 2,3D and 2,4D acids are the least aromatic systems, which means that they will be the most reactive systems. Unsubstituted acid (phenoxyacetic acid) is the most stable system. Comparing both monosubstituted acids with respect to phenoxyacetic acid, it can be seen that the degree of disturbance of the aromatic system is greater in the case of 2,3D than in the case of 2,4D. The decrease in aromaticity in a series of phenoxyacetic acids based on the analysis of infrared and Raman spectra can be summarised in the following series: PA→4CPA→2CPA→MCPA→2,4D→2,3D.

#### 3.2.2. UV-VIS Study

**Figure 9:**
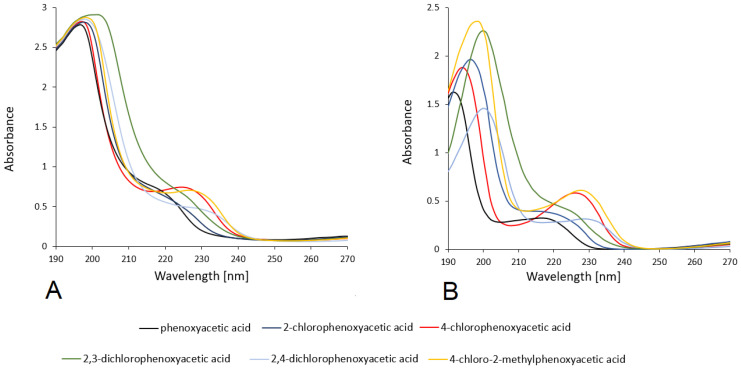
The experimental of UV spectra for chlorophenoxyacids measured in methanol (**A**) and water solutions (**B**).

UV spectra of the tested compounds were recorded in methanol and water solutions with concentrations of 5 × 10^−5^ mol/dm^3^. The spectra of the tested compounds were calculated using the TD-DFT/6-311++G(d,p) method using the CPCM solvent model (water and methanol). The spectra are presented in Figure 9. Table 4 shows the values of the absorption maxima λmax for the tested acids, measured experimentally in aqueous and methanolic media and theoretically calculated, along with the assignment. In the UV electronic spectra in the range of 190–270 nm. In the spectrum of phenoxyacetic acid recorded in methanol, two absorption maximum (λ_max_ = 197 nm and 217 nm) are observed, which corresponding to electronic transitions in the aromatic system π→π*. Modeling of the theoretical spectrum indicates that this band is responsible for electronic transitions between the H-1→LUMO, H-1→L+1, H-1→L+2 and HOMO→L+1 levels for first band and H−1→LUMO, H−1→L+1, H−1→L+2 levels for second band. In aqueous solution, the maximum value of those band are observed at 192 nm and 216 nm (electronic transitions are the same as in methanol solution). In the electronic spectra of chlorosubstituted phenoxyacetic acid, the bands (both in aqueous and methanolic solutions and theoretically modeled) are located at higher wavelengths than in the acid (hypsochromic effect).

The exception is 4CPA acid, the absorbance maximum of which is located at the same value as in the spectrum of PA acid. The highest calculated electronic transition energy for the observed absorption maxima is 6.3378 eV and 6.6339 eV (in aqueous and methanol solutions, respectively). The values of absorbance maxima in aqueous solutions are slightly lower than in methanol solutions, which is related to solvent effects. However, these values in the modeled theoretical spectra in water and methanol solvents are the same. In the tested herbicides (theoretical calculations in aqueous and methanol solutions), there is an increase in the energy of the π→π* electron transition in the series 2CPA→2,4D→MCPA→2,3D→PA→4CPA.

##### 3.2.3. ^13^CNMR and ^1^HNMR

The values of ^1^HNMR and ^13^CNMR chemical shifts recorded for the acids derived from phenoxyacetic acid in DMSO and calculated using the B3LYP/6−311++(d,p) method using the model DMSO solvent (CPMC method) are listed in Table 5. The analysis of chemical shift values was used to evaluate the impact of substituting the chlorine atom/atoms on the change in the electronic charge distribution in the tested molecules. In all chlorinated derivatives of phenoxyacetic acid, a decrease in the value of chemical shifts of the C1 atom was observed, which is related to the increase in electron density around this atom. The values of chemical shifts of the C2 carbon increase significantly in derivatives in which substituents to this atom are substituted in the aromatic ring, which was observed in the 2CPA, 2,3D, 2,4D, and MCPA derivatives. This is related to a significant decrease in the electron density around this atom. In the case of 4CPA, the decrease in electron density around the C2 atom with respect to the unsubstituted PCA acid is small. Substituting chlorine in the aromatic ring in position 2 (2CPA, 2,4D) causes an increase in the electron density around the C3 carbon, which results in a decrease in the value of 13CNMR chemical shifts in these compounds in relation to the unsubstituted acid. In the remaining analysed compounds, a decrease in electron density around the C3 carbon was observed. The electron density around the C4 atom in all phenoxyacetic acid derivatives decreases, with the greatest effect observed in the derivatives in which the chlorine atom is substituted at the C4 position (4CPA, 2,4D, MCPA). The change in the chemical shift values of the C5 carbon indicates an increase in the electron density around this atom in all chlorine derivatives relative to unsubstituted phenoxyacetic acid. The electron density around the C6 carbon atom in substituted PA derivatives generally increases with the exception of 4-chlorophenoxyacetic acid. A detailed analysis of changes in electron density in the tested compounds indicates that the increase in the disturbance of electron charge distribution occurs in the tested series in the direction of: PA→4CPA→2CPA→2,4D→2,3D→MCPA. These results are consistent with the theoretical data, calculated values of the NBO electron charge, and the results of other experimental studies. The changes in electron density around the aliphatic carbon C7 are insignificant. A similar conclusion can be made in the case of the carbons of the carboxyl group of acids marked as C8. The values of C8 chemical shifts decrease in chlorine derivatives of PA acid under the influence of chlorine substitution in the aromatic ring, which indicates an increase in electron density. However, this is a slight increase and occurs in the series: PA→ 4CPA→MCPA→2CPA→2,4D→2,3D.

The analysis of chemical shifts of protons in the aromatic system can be used to assess the aromaticity of the tested chemical compound. The increase in the value of proton chemical shifts in the ^1^HNMR spectrum indicates an increase in the aromaticity of the ring. In the tested phenoxyacetic acid derivatives, the aromatic protons were substituted with a chlorine atom or chlorine atoms and a methyl group, which does not allow for a thorough analysis of the aromaticity of the tested systems based on observations of changes in chemical shifts in the tested series of compounds. The values of chemical shifts of the H5 proton observed in the spectra of 2CPA, 2,3D and MCPA acid decrease compared to the unsubstituted acid, which indicates a decrease in the aromaticity of these systems. In the case of the H6 proton, the chemical shift values are slightly higher in the 2CPA, 2,3D and 2,4D derivatives, which indicates an increase in the aromaticity of the ring. Generally, based on the analysis of ^1^HNMR proton spectra, it can be concluded that the MCPA acid has the lowest aromaticity among the tested systems. The aromaticity of 2CPA and 4CPA acids is similar to that of unsubstituted chlorophenoxyacetic acid PA. Disubstituted acids have slightly lower aromaticity. A slight decrease in the electron density around the protons (H7 and H8) of aliphatic carbon in the tested PA acid derivatives was observed, which is manifested by an increase in the value of chemical shifts in the ^1^HNMR spectrum.

### 3.3. Results of Reactivity Studies

**Figure 10:**
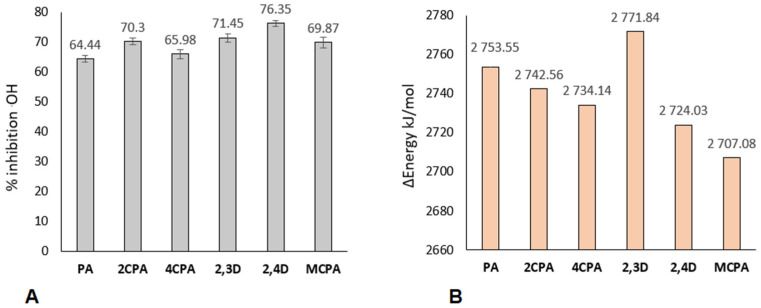
(**A**) Percentage of inhibition of hydroxyl radical (**B**) Energy reaction herbicides with hydroxyl radical calculated in B3LYP/6-311++G(d,p) (water solution model od CPCM).

Figure 10A shows the results of experimental tests on the reactivity of herbicides with the hydroxyl radical .OH. The degree of inhibition of the hydroxyl radical in reactions with phenoxyacetic acids was taken as a measure of reactivity. It was observed that all of the tested chlorinated derivatives of phenoxyacetic acid are characterised by higher reactivity towards the hydroxyl radical than PA acid. 2,4D acid has the highest ability to react with the hydroxyl radical. Generally, disubstituted acids are characterised by a higher ability to remove the hydroxyl radical. These compounds are characterised by lower aromaticity than monosubstituted acids and are therefore more reactive. It was observed that an increase in the destabilisation of the electron system of a molecule increases its reactivity. The reaction of the hydroxyl radical with phenoxyacetic acids may produce the phenoxy radical and glycolic acid [43]. The energy effects of this reaction were calculated using the B3LYP-6-3121++G(d,p) method, taking into account the aqueous environment (CPCM computational model). The calculation results are presented in Figure 10B.

The computational data show that the place of substitution of the chlorine atom/atoms in the aromatic ring of phenoxyacetic acid influences the susceptibility of a molecule to reactions with the hydroxyl radical. All molecules except 2,3D show higher reactivity towards the hydroxyl radical than phenoxyacetic acid. MCPA has the highest reactivity.

**Figure 11:**
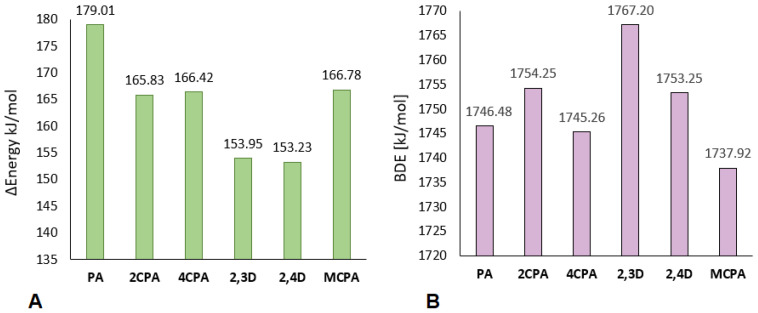
Deprotonation energy (**A**) and Bond Dissotiation Energy (**B**) for chlorophenoxy acids calculated in B3LYP/6-311++G(d,p) (water solution model od CPCM).

Chloroderivatives of phenoxyacetic acid are also characterised by a lower deprotonation energy than unsubstituted phenoxyacetic acid in the ring (Figure 11A). PA acid derivatives disubstituted with chlorine atoms are characterised by a similar deprotonation ability, as evidenced by similar deprotonation energy values (about 150 kJ/mol). Acids substituted with one chlorine atom, i.e., 2CPA, 4CPA and MCPA, are characterised by higher deprotonation energy, but for both of them this energy is at a similar level (about 165 kJ/mol). The 2,3-dichlorophenoxyacetic acid has the highest proton binding energy of the carboxyl group (BDE), while MCPA has the lowest (Figure 11B). Acid molecules in which the chlorine atom is substituted in position 2 in the aromatic ring (2CPA, 2,3D and 2,4D), i.e., closest to the carboxyl group, are characterised by a higher BDE energy value than the unsubstituted acid—PA. However, molecules in which the chlorine atom is substituted further from the carboxyl group are characterised by a lower BDE value than phenoxyacetic acid.

### 3.4. Antibacterial Activity

Soil microbes serve as reliable indicators of soil health, contributing substantially to the functioning of the agroecosystem by facilitating nutrient cycling by converting unavailable forms of phosphorus (*B. megaterium*, *P. aeruginosa*) and potassium (*B. megaterium*) into available forms [58,59] promoting soil microbiome resilience, and exerting natural control over plant pests and pathogens [60]. Therefore, it is important that herbicides do not have a negative impact on soil bacteria, the task of which is to stimulate plant growth, increase the yield of plants with high potassium and phosphorus requirements, and increase the bioavailability of these essential nutrients.

**Figure 12:**
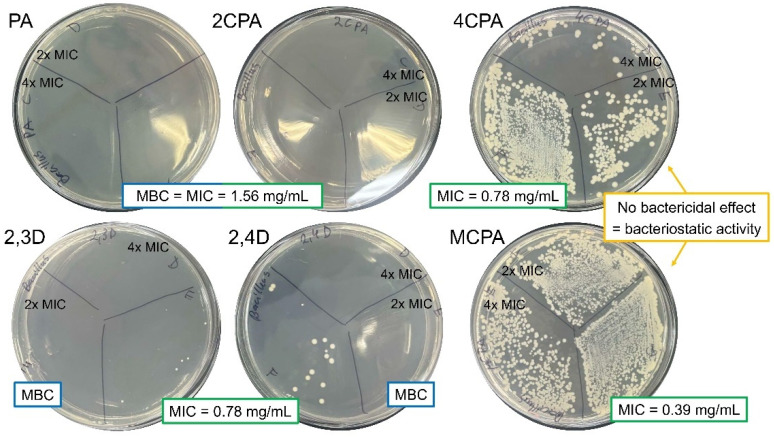
Bactericidal and bacteriostatic activity of tested herbicides against *Bacillus megaterium*.

**Figure 13:**
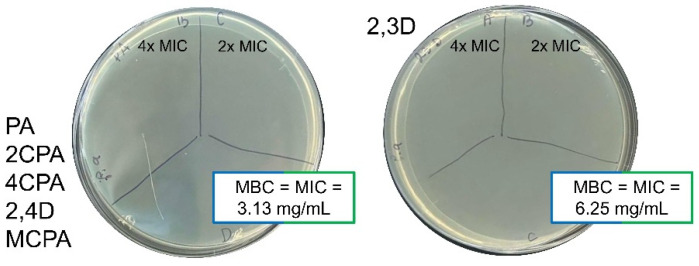
Bactericidal activity of tested herbicides against *Pseudomonas aeruginosa*.

The evaluation of the antibacterial activity of herbicides was carried out by determining the MIC and MBC values. *P. aeruginosa* is generally more resistant to treatment with tested herbicides compared to *B. megaterium* (Table 6, Figure 12 and Figure 13). The MIC value determined for all herbicides against *P. aeruginosa* was 3.13 mg/mL, except 2,3D, with MIC = 6.25 mg/mL. The MBC values for the six herbicides were equal to the MIC values of the corresponding herbicides [Figure 13], indicating their bactericidal activity against *P. aeruginosa*. The only compounds with bacteriostatic activity against *B. megaterium* were MCPA and 4CPA, for which the MIC, 2xMIC, and 4xMIC concentrations resulted in inhibition of bacterial growth. It is worth comparing the antibacterial properties of the compounds tested with the antibiotics that exhibit antibacterial activity up to 100 and 10^6^-fold higher, respectively, against *P. aeruginosa* and *B. megaterium* (Table 6). Therefore, the six herbicides cannot be considered antibacterial compounds.

### 3.5. Cytotoxicity of Herbicides

Studies on the potential impact of phenoxy herbicides, such as 2,4-D and MCPA, on cancer cells have already yielded conflicting and ambiguous results. Thus, we used DU-145, a prostate cancer cell line, to evaluate how 2,4-D and MCPA affect cancer cells. A link between exposure to phenoxy herbicides and an increased risk of certain cancers, including prostate cancer, was already suggested [21,61,62]. However, other studies have found no significant association or have shown only weak links [63]. Confounding factors, such as exposure to other chemicals and differences in study methods, make it difficult to draw definitive conclusions.

**Figure 14:**
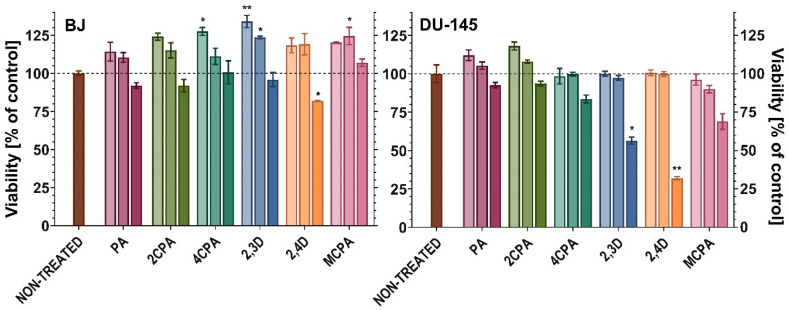
Viability of BJ (left) and DU-145 (right) cells after 24 h of incubation with 6 herbicides at a concentration of 25, 100 and 400 µg/mL (the higher concentration of the herbicide the darker color of bar). Cell viability (expressed as percent of the control not treated) means ± SD are plotted. The results represent statistical significance in relation to the untreated control (Kruskal-Wallis test, *—*p* < 0.05, **—*p* < 0.001).

To ensure that pesticide treatment did not cause dramatic changes in normal cell viability or does not lead to increased viability of cancer cells, respectively, normal BJ fibroblast normal cells and DU-145 prostate cancer cells were treated with the tested herbicides (Figure 14).

Concentration-dependent (25, 100 and 400 µg/mL) cytotoxicity was evaluated using water-soluble tetrazolium WST-1, which cleaves to formazan by mitochondrial dehydrogenases [64].

Against normal cells, statistically significant cytotoxicity was observed only after treatment with the highest concentration (400 µg/mL) of 2,4D, where cell viability decreased by 18.12% compared to the untreated control sample. Interestingly, the concentration of 25 µg/mL of 4CPA, 2,3D and 100 µg/mL of 2,3D and MCPA induced the growth of normal BJ cells ranging from 23.62% to 34.03%. Only treatment with the highest concentration of 2,3D and 2,4D decreased the viability of DU-145 prostate cancer cells, respectively, by 43.54% and 68.06%. The results revealed that PA and 2CPA have no influence on normal fibroblasts at any used concentration. Prostate cancer cell viability is not significantly stimulated by any of the herbicides tested.

## 4. Conclusions

The physicochemical properties and biological activity of chemical compounds are determined by their molecular structure and the distribution of electronic charge in the molecules. By introducing substituents into the aromatic ring, the electronic structure of the ligands and thus their biological activity can be changed. The reactivity of aromatic compounds depends on the type of substituents attached to their ring. Substituents can cause mesomeric, inductive, activating and deactivating effects, which are related to the change in the electronic charge distribution in the aromatic ring. The stabiliwation or disturption of the π-electron system of an aromatic ring can be examined using a number of different methods. These include spectroscopic methods (FT-IR and Raman FT-Raman infrared spectroscopy, ^1^HNMR and ^13^CNMR nuclear magnetic resonance spectroscopy, UV-VIS spectrophotometry), X-ray diffraction (observation of changes in the structure—bond lengths and angle values in the ring), calculations of aromaticity indices based on structural parameters, magnetic properties and reactivity), calculations of electronic charge distribution using quantum chemical methods for optimised structures of the tested compounds (e.g., NBO (Natural Bond Orbital)). These methods are complementary—the results obtained using different methods lead to similar conclusions, which were also observed in this work. The substitution of the chlorine atom/atoms in the aromatic ring of phenoxyacetic acid caused significant changes in the distribution of electronic charge in the tested molecules.

Along with the change in the electron density of the atoms in the tested molecules, their reactivity and biological activity changed. Certain relationships have been observed between the reactivity and biological activity of herbicide molecules and changes in their molecular structure caused by the appearance of substituents in the aromatic ring.

Studies on fibroblasts and prostate cancer cell lines have shown that derivatives of phenoxyacetic acid disubstituted with chlorine atoms (2,3D, 2,4D and MCPA) are characterised by the highest cytotoxicity. These compounds are characterised by reduced stability in the electronic charge distribution (low aromaticity), and at the same time they are characterised by high reactivity and biological activity. Monosubstituted derivatives and pure phenoxyacetic acid are compounds with increased electronic stability (aromaticity) and lower reactivity. They are characterised by reduced cytotoxicity compared to disubstituted phenoxyacetic acid derivatives. What is important when it comes to the use of herbicides is their impact on soil microorganisms, which are necessary to maintain proper soil environmental conditions. Studies conducted on the bacteria *B. megaterium* and *P. aeruginosa* did not show a significant biocidal effect of the tested herbicides compared to the effect of gentamicin. However, it was reported that the minimum inhibitory concentration (MIC) against *Bacillus megaterium* in the case of disubstituted derivatives of 2,3D and 2,4D herbicides is half of that in the case of phenoxyacetic acid and the monosubstituted derivative 2CPA, and in the case of MCPA up to 5 times lower.

The conducted research has shown that under the influence of substitution with a chlorine atom/atoms in the aromatic ring of phenoxyacetic acid, the distribution of electronic charge in the molecule changes. There is a destabilisation of the electronic charge distribution of the aromatic ring, a decrease in aromaticity and an increase in reactivity. Disubstituted molecules are characterised by higher reactivity and biological activity than monosubstituted derivatives. In the studied group of compounds, however, no significant correlations were found between parameters describing the structure and their antimicrobial activity and cytotoxicity (the value of the correlation coefficient R2 was below 0.7). The relationships between parameters such as aromaticity indices, deprotonation energies, frontal orbital energies and reactivity descriptors, chemical shifts in NMR spectra and MIC, MBC, cytotoxicity level were analyzed. Correlation analysis showed that the relationships between these parameters are statistically insignificant. In the case of this group of compounds, the activity of these compounds can only be assessed descriptively in the context of changes in their structure. In order to deepen the QSAR analysis, more biological tests should be performed with the extension to a larger group of organisms and cell lines.

## Figures and Tables

**Table 1:** Frontier orbitals energy values, reactivity descriptors and geometric aromaticity.

	PA	2CPA	4CPA	2,3D	2,4D	MCPA
Energy [eV]	−14,571.57	−27,078.42	−27,078.52	−39,585.24	−39,585.33	−28,148.71
	**−14,571.91**	**−27,078.81**	**−27,078.87**	**−39,585.64**	**−39,585.72**	**−28,149.05**
Dipole moment [D]	2.48	3.97	3.04	4.97	4.01	2.99
	**3.69**	**5.83**	**4.33**	**7.21**	**5.78**	**4.16**
E_HOMO_ [eV]	−9.2668	−8.2034	−8.0899	−7.9120	−7.9340	−8.0755
	**−9.2603**	**−8.2034**	**−8.0897**	**−7.9226**	**−7.9346**	**−8.0744**
E_LUMO_ [eV]	−5.1859	−4.8077	−5.1767	−4.5391	−4.7868	−5.0700
	**−5.1870**	**−4.8134**	**−5.1789**	**−4.5438**	**−4.7922**	**−5.0725**
Δ = E_LUMO_ − E_HOMO_ [eV]	4.0809	3.3957	2.9133	3.3729	3.1473	3.0055
	**4.0733**	**3.3900**	**2.9108**	**3.3788**	**3.1424**	**3.0020**
Ionisation potential I = −E_HOMO_	9.2668	8.2034	8.0899	7.9120	7.9340	8.0755
	**9.2603**	**8.2034**	**8.0897**	**7.9226**	**7.9346**	**8.0744**
Electron affinity A = −E_LUMO_	5.1859	4.8077	5.1767	4.5391	4.7868	5.0700
	**5.1870**	**4.8134**	**5.1789**	**4.5438**	**4.7922**	**5.0725**
Electroegativity Χ = (I + A)/2	7.2264	6.5056	6.6333	6.2256	6.3604	6.5728
	**7.2237**	**6.5084**	**6.6343**	**6.2332**	**6.3634**	**6.5735**
Chemical potential μ = −(I + A)/2	−7.2264	−6.5056	−6.6333	−6.2256	−6.3604	−6.5728
	**−7.2237**	**−6.5084**	**−6.6343**	**−6.2332**	**−6.3634**	**−6.5735**
Chemical hardness η = (I − A)/2	2.0404	1.6979	1.4566	1.6864	1.5736	1.5027
	**2.0366**	**1.6950**	**1.4554**	**1.6894**	**1.5712**	**1.5010**
Chemical softness S = 1/(2η)	0.2450	0.2945	0.3433	0.2965	0.3177	0.3327
	**0.2455**	**0.2950**	**0.3435**	**0.2960**	**0.3182**	**0.3331**
Electrophilicity index ω = μ^2^/2η	12.7964	12.4635	15.1037	11.4910	12.8539	14.3741
	**12.8107**	**12.4955**	**15.1208**	**11.4988**	**12.8860**	**14.3940**
Aromaticity indices						
HOMA	0.983	0.978	0.986	0.969	0.979	0.974
	**0.980**	**0.979**	**0.986**	**0.973**	**0.981**	**0.971**
	*0.987*	*0.711*	*0.972*	*0.990*	*0.981*	*0.912*
GEO	0.005	0.009	0.007	0.013	0.012	0.014
	**0.004**	**0.008**	**0.006**	**0.011**	**0.010**	**0.014**
	*0.009*	*0.986*	*0.014*	*0.009*	*0.016*	*0.071*
EN	0.012	0.013	0.008	0.017	0.009	0.012
	**0.016**	**0.014**	**0.009**	**0.016**	**0.009**	**0.014**
	*0.004*	*0.001*	*0.014*	*0.001*	*0.003*	*0.016*
I6	96.16	95.01	95.73	93.97	94.49	93.95
	**96.69**	**95.48**	**95.98**	**94.56**	**94.78**	**93.77**
	*94.91*	*71.66*	*93.84*	*95.02*	*93.45*	*85.75*
Aj	0.998	0.996	0.997	0.994	0.995	0.994
	**0.998**	**0.997**	**0.997**	**0.995**	**0.995**	**0.993**
	*0.996*	*0.870*	*0.993*	*0.996*	*0.993*	*0.968*
BAC	0.930	0.920	0.928	0.913	0.917	0.903
	**0.939**	**0.927**	**0.935**	**0.924**	**0.923**	**0.905**
	*0.900*	*0.525*	*0.907*	*0.906*	*0.885*	*0.749*

Standard font—values calculated in vacuum; bold—values calculated in water; italics—values calculated for experimental bond lenghts.

**Table 2:** Wavenumbers (cm^−1^), intensities and assignments of bands occurring in the IR (KBr, ATR and DFT) and Raman spectra of phenoxyacetic acid, 2-chlorophenoxyacetic acid and 4-chlorophenoxyacetic acid.

Phenoxyacetic Acid					2-Chlorophenoxyacetic Acid					4-Chlorophenoxyacetic Acid					Assignments	
Experimental			Calculated		Experimental			Calculated		Experimental			Calculated			
FTIR_KBR_	FTIR_ATR_	Raman	IR	Inten.	FTIR_KBR_	FTIR_ATR_	Raman	IR	Inten.	FTIR_KBR_	FTIR_ATR_	Raman	IR	Inten.		
3443 m			3690	91.51	3431 m			3690	95.09	3425 m			3690	96.81		ν(OH)
3060 m	3064 vw	3060 s	3136	1.17	3063 m		3060 s	3144	4.28	3054 m		3066 vs	3145	4.12	2	ν(CH)
3036 m		3038 w	3133	8.95	3039 m			3137	7.71				3131	2.95	20a	ν(CH)
3012 m	3010 vw	3013 vw	3125	22.10	3003 m		2999 w	3125	10.63				3128	3.55	20b	ν(CH)
2921 m	2920 w	2923 w	2973	13.31	2920 m	2918 vw	2920 m	2983	11.19	2925 m		2916 m	2976	12.43		ν_as_CH_2_
2799 m	2804 vw		2940	33.02	2792 m	2795 xw		2947	31.92	2781 w		2788 w	2942	33.57		ν_s_CH_2_
1736 s	1732 m				1742 s	1742 m				1743 s	1743 s					νC=O
1703 s	1702 s		1814	310.86	1712 s	1711 m		1819	295.01	1708 m	1706 m		1815	312.41		νC=O
1599 m	1597 w	1598 m	1609	70.23				1597	45.82	1647 w			1604	27.11	9a	β(CH), ν(CC)
1585 m	1585 w	1585 m	1592	30.04	1589 m	1587 w	1589 m	1582	6.23	1599 w		1598 m	1584	13.86	9b	β(CH), ν(CC),
1498 s	1498 m	1487 vw	1495	95.42	1485 vs	1485 s		1487	111.86	1494 s	1494 s	1491 w	1491	189.33	18a	β(CH), ν(CC)
1470 w	1470 w	1459 w	1458	17.31									1444	2.37	18b	β(CH), ν(CC)+ β CH_2_
1437 s	1437 m	1439 m	1452	39.72	1447 m	1446 w		1445	67.46	1427 s	1427 m	1431 m	1454	73.85		βCH_2_
								1402	11.07	1400 m	1400 m	1409 w	1398	12.42		γCH_2_
1374 w	1374 m	1381 w	1399	11.26	1383 m	1374 w				1383 m	1378 m	1389 w				γCH_2_, νCC_al_,
1338 w	1341 w	1339 w	1330	17.21						1365 w	1364 w		1296	5.03	3	β(CH), ν(CC)
1310 m	1310 w	1310 vw	1305	33.09	1313 m			1303	49.38	1297 m	1297 m	1285 vw	1300	73.60	8b	β(CH), ν(CC)
1300 m	1307 w	1306 vw	1282	4.97				1288	11.89				1283	0.74		β(OH), γCH_2_
1289 m	1289 w	1289 w	1248	275.65	1290 s	1290 m		1273	98.39				1249	289.97		β(CH), RC-O, γCH_2_
1266 m	1267 w		1233	1.93				1253	2.07				1234	2.15		τCH_2_
					1245 vs	1245 s	1255 w			1246 vs	1246 vs	1253 w				α(CCC), νC-O
1235 vs	1229 vs									1239 vs	1238 vs	1231 vw	1125	260.30		α(CCC), νC-O
1184 m	1185 w	1185 vw	1173	10.40				1163	14.50	1194 vs	1194 s		1171	12.15	8a	β(CH)
1172 m	1172 w	1174 w	1156	1.77	1167 m	1167 w	1168 m	1119	26.35				1107	36.83	14	β(CH)
1158 m	1160 m	1160 w	1124	238.79	1137 m	1137 w	1142 w	1082	305.35	1137 s	1137 s					νC-OH, βCH_2_, βOH,
1093 s	1093 m		1085	178.43	1090 s	1089 s	1091 w			1099 m	1100 m	1098 m				β(CH), βOH,
1073 m	1073 m	1075 w	1076	106.13	1051 m	1052 m				1078 m	1079 m	1078 w	1072	233.93		β(CH), νO-CH_2_
					1037 m	1038 m	1039 s									β(CH)
1022 w	1022 w	1028 w	1023	2.08	1015 w			1048	7.54	1018 w	1018 w		1002	15.64	19a	β(CH), ν(CC)
997 w	995 w	996 s	991	0.94				1040	43.02	996 w	995 w	1004 vw			12	α(CCC)
961 w	961 w	963 w	967	0.14						956 m	950 m		949	0.11	5	γ(CH)
930 w	931 w	934 vw	949	0.14	937 m	940 m	932 w	958	0.19	927 m		920 vw	916	0.18	10b	γ(CH)
913 w	915 w	914 w	885	2.47	913 m	913 m				918 m	913 m		885	4.56		βO-CH_2_
885 w	888 w	884 vw	875	6.97				919	2.42	878 m	878 m		821	57.00	17b	γ(CH)
834 m	835 w	829 w	820	22.91	840 m	840 m	841 w	827	13.21				825	3.77	6a	α(CCC)
821 m	823 w	823 w	809	0.36	821 w	823 w		834	0.35	801 s	803 vs		791	12.02	10a	γ(CH)
755 s	755 s	758 vw	749	68.56	756 vs	755 vs	756 vw	745	66.78	755 vw	755 w				11	γ(CH)
					715 m	716 s	706 w			721 vw	717 w					α(CCC)
690 m	690 m		688	34.98	667 w	667 w	661 w			685 s	685 m	698 w	711	1.37	4	γ(CH)
			643	26.91												γCH_2_, βOCO
								697	31.63				678	67.30	6a	α(CCC)
			633	75.53						649 w	654 w	635 w	634	84.84		γOH
613 w	615 vw	614 w	616	2.22				632	23.06				633	0.27	6b	α(CCC)
578 w		571 vw	551	18.99	586 w		584 vw			578 w			599	13.75		α(CCC) + βOCO
530 w		520 vw	519	50.95				632	75.83				510	56.63		γOH, τCH_2_
510 w		502 vw	502	3.98				557	2.23	506 vw			505	1.28	16b	γ(CH)+γOH

α(CCC)—deformation of ring, ν—streching, β—deforming in plane, γ—deforming out of plane, s—symmetric, as—asymmetric, al—aliphatic atoms, intensities: vs—very strong, s—strong, m—medium, w—weak.

**Table 3:** Wavenumbers (cm^−1^), intensities and assignments of bands occurring in the IR (KBr, ATR and DFT) and Raman spectra of 4-chloro-2-methylphenoxyacetic acid, 2,3-dichlorophenoxyacetic acid and 2,4-dichlorophenoxyacetic acid.

4-Chloro-2-Methylphenoxyacetic acid					2,3-Dichlorophenoxyacetic acid					2,4-Dichlorophenoxyacetic acid					Assignments	
Experimental			Calculated		Experimental			Calculated		Experimental			Calculated			
FTIR_KBR_	FTIR_ATR_	Raman	IR	Inten.	FTIR_KBR_	FTIR_ATR_	Raman	IR	Inten.	FTIR_KBR_	FTIR_ATR_	Raman	IR	Inten.		
3440 m			3647	9658	3445 vs			3689	99.09	3430 m			3689	100.24		ν(OH)
3425 m					3424 vs										1	ν(CH)
3054 m	3053 w	3058 m	3146	4.02	3097 w	3098 w	3096 m	3148	2.46				3152	1.41	2	ν(CH)
							3078 s	3144	3.60	3073 m		3083 vs	3149	1.85	20a	ν(CH)
			3125	2.99	2987 w	2981 w		3119	5.30	2978 m	2979 w	2978 vs	3136	3.79	20b	ν(CH)
			3023	10.09												βCH(CH_3_)
							2946 m					2950 s				ν(CH)
2925 m	2929 w	2930 vs	2976	12.03	2920 w	2918 w	2920 m	2985	10.14			2921 w	2983	10.54		ν_as_CH_2_
			2974	18.94												νC-H(CH_3_)
			2942	36.01	2794 w	2794 w		2948	31.47	2755 w	2758 w		2946	32.51		ν_s_CH_2_
1743 s	1743 s	1739 w	1813	300.52	1743 s	1743 m	-	1819	287.72	1735 s	1729 s		1856	295.96		νC=O
1708 m	1706 m	1704 w			1708 s	1706 m	-			1709 m						νC=O
1637 w	1636 w	1640 w	1600	9.25	1634 m	1635 w				1628 m	1635 w		1591	13.81	9a	β(CH), ν(CC)
1599 w	1597 w	1599 vs	1587	1.86	1582 m	1575 w	1575 s	1582	111.50	1585 w	1585 w	1593 s	1604	8.27	9b	β(CH), ν(CC),
1494 s	1494 w	1497 w	1490	173.35									1482	212.96	18a	β(CH), ν(CC)
1450 w	1449 w	1449 w	1452	72.69				1445	194.73	1449 m	1449 m	1445 s	1451	116.57		βCH_2_
			1448	8.42												τCH_3_
			1397	17.91	1460 s	1460 m	1458 m			1478 s	1477 s	1481 w			18b	β(CH), ν(CC)+ β CH_2_
1427 s	1427 m	1433 m	1398	13.15	1430 m	1429 s	1428 m	1398	7.81	1429 m	1429 m	1424 w	1398	11.89		γCH_2_
1400 m	1400 w	1403 w	1393	1.80												βCH_3_
1383 m	1379 w	1378 m			1383 m		1392 w			1385 m	1391 w	1393 w	1385	14.58	15	γCH_2_, νCC_al_,
1365 w	1364 w	1364 w						1297	72.41	1362 vw	1361 w	1371 w	1295	111.17	3	β(CH), ν(CC)
			1305	56.28												α(CCC)
					1307 w	1307 m	1311 w			1310 m	1310 m	1310 m	1263	164.23	8b	β(CH), ν(CC)
													1286	1.00		β(OH), γCH_2_
1297 m	1297 m	1297 m	1284	2.52	1287 m	1283 m	1282 m	1236	2.27							β(CH), RC-O, γCH_2_
			1271	10.10									1251	44.22		β(CH)
		1257 m	1232	2.20	1270 m	1272 s				1260 m	1260 m	1258 s	1236	2.31		τCH_2_
1246 vs	1246 vs				1248 m	1248 vs	1253 m									α(CCC), νC-O
1240 vs	1238 vs		1247	226.42						1232 vs	1231 vs	1234 m				α(CCC), νC-O
1194 vs	1194 s	1195 m	1187	60.33	1195 w	1195 w	1195 m	1194	3.53				1152	1.01	8a	β(CH)
1137 s	1137 s	1130 w	1143	0.24	1168 w	1171 w	1173 m	1160	4.79						14	β(CH)
								1108	94.82	1158 w	1160 w	1160 s	1123	202.77		νC-OH, βCH_2_, βOH,
		1115 m	1114	407.98	1113 m	1112 m	1109 m			1105 m	1105 m	1105 m				β(CH), βOH,
1099 m	1100 w	1103 m	1072	159.43	1088 s	1085 s				1093 s	1092 s		1082	343.92		β(CH), νO-CH_2_
1078 m	1079 m	1082 m			1051 m	1049 w	1049 vs			1048 w	1048 w					β(CH)
1018 w	1018 w	1023 w	1095	10.76						1027 w	1027 w	1026 w	1096	65.39	19a	β(CH), ν(CC)
			1042	1.65												τCH3
			997	11.27												γCH3
995 w	995 w	1000 w			1003 vw			1043	42.00				1046	7.92	12	α(CCC)
								1012	0.29				1013	0.35		βCH_2_
956 w	950 w	960 w						947	0.26						5	γ(CH)
927 w		928 s	908	0.80									916	0.04	10b	γ(CH)
918 w	913 m		884	7.21	918 w	915 m	915 m	889	9.56	915 w	915 m		885	5.15		βO-CH_2_
888 w	889 w	899 w			895 m	895 s				895 w	895 m	893 s			17b	γ(CH)
878 w	878 m	888 w	873	19.37	861 vw	861 w				872 w	871 m				7 a	α(CCC)
			870	14.18				874	40.69				873	17.36	17a	γ(CH)
										837 w	837 w	840 vs	843	8.06	6 a	α(CCC)
801 s	803 vs	799 m	792	39.72						794 m	794 s	805 w	795	39.38	10a	γ(CH)
755 vw	755 w				767 m	766 s		762	48.54	759 vw	758 w				11	γ(CH)
					736 vw	736 w		731	7.93	721 m	721 m	718 s	743	94.39		α(CCC)
685 m	685 m	680 m	711	0.01	709 w	711 m				697 w	697 m	698 w	730	0.53	4	γ(CH)
649 w	654 w	654 w	635	84.75						646 w	646 m	647 s				γOH
616 vw	613 w		666	41.62	613 w	613 w	611 s	668	31.46	596 w		594 m	656	19.65	6b	α(CCC)
578 vw		576 m	609	18.02	568 w			632	77.45	555 w		560 m	608	21.38		α(CCC) + βOCO
554 w			565	5.77											16b	γ(CH)
506 w		508 w	509	40.03									647	83.80		γOH, τCH_2_
								568	0.01				563	6.69	16a	γ(CH)
474 vw			441	3.22	474 w			502	11.63	469 w						γ(CH)+γOH

α(CCC)—deformation of ring, ν—streching, β—deforming in plane, γ—deforming out of plane, s—symmetric, as—asymmetric, al—aliphatic atoms, intensities: vs—very strong, s—strong, m—medium, w—weak.

**Table 4:** The experimental and theoretical (TD-B3LYP/6-311++G(d,p)) of maximum absorbance on UV spectra for chlorophenoxyacids measured in methanol and water solutions.

	Water Solution C = 5 × 10^−5^ mol/dm^3^				Methanol Solution C = 5 × 10^−5^ mol/dm^3^				Assignments	
	Experimental λ_max_ [nm]	Theoretical λ_max_ [nm]	Oscillator Strength (f)	Band Gap [eV]	Experimental λ_max_ [nm]	Theoretical λ_max_ [nm]	Oscillator Strength (f)	Band Gap [eV]		
**PA**	192	205	0.1330	6.1638	197	205	0.1323	6.1612	H−1→LUMO H−1→L+1 H−1→L+2 HOMO→L+1	π→π*
	216	216	0.0904	5.7355	217	216	0.0898	5.7351	HOMO→L+2 H−1→ L+1 H−1→LUMO	π→π*
**2CPA**	196	205.5	0.2421	6.0616	198	205.5	0.2421	6.0616	H−1→LUMO H−1→L+1 H−1→L+2 HOMO→L+1 HOMO→L+2	π→π*
	219	219.5	0.0585	5.6395	222	219.5	0.0512	5.6395	HOMO→L+3 HOMO→L+2 HOMO→LUMO H−1→LUMO	π→π*
**4CPA**	194	196	0.3547	6.3378	197	196	0.3511	6.6339	H−1→LUMO H−1→L+1 H−1→L+2 HOMO→L+1	π→π*
	226	224	0.2174	5.5260	225	224	0.2157	5.5263	H−1→LUMO HOMO→L+1 HOMO→L+2	π→π*
**2,3D**	200	203.5	0.5249	6.1223	201	203.5	0.5267	6.1205	H−1→LUMO H−1→L+1 HOMO→LUMO HOMO→L+3	π→π*
	220	222	0.024	5.5861	221	222	0.028	5.5856	HOMO→L+1 HOMO→L+2 H−1→LUMO H−1→L+2	π→π*
**2,4D**	200	204.5	0.6435	6.0795	198	204.5	0.6420	6.0772	H−1→LUMO H−1→L+2 HOMO→L+1	π→π*
	229	224	0.0812	5.5007	227	224	0.0796	5.5000	H−1→L+1 HOMO→L+2 HOMO→L+3	π→π*
**MCPA**	198	204.5	0.2453	6.1104	198	205	0.2441	6.1086	H−1→LUMO H−1→L+1 H−1→L+2 HOMO→L+1 HOMO→L+2	π→π*
	228	226	0.1658	5.4883	227	226	0.1651	5.4878	H−1→LUMO HOMO→L+1 HOMO→L+2	π→π*

**Table 5:** The experimental and theoretical (GIAO method calculated in B3LYP/6-311++G(d,p)) ^1^HNMR and ^13^CNMR chemical shifts for chlorophenoxyacids.

Atom	PA		2CPA		4CPA		2,3D		2,4D		MCPA	
	Exp	Calc	Exp	Calc	Exp	Calc	Exp	Calc	Exp	Calc	Exp	Calc
C1	157.81	164.20	153.36	158.98	156.73	162.80	154.85	160.86	152.48	158.56	154.94	160.92
C2	114.47	121.53	128.23	133.83	116.34	123.31	128.31	132.70	127.96	135.99	128.61	136.41
C3	129.54	134.44	130.24	135.43	124.87	134.33	132.56	146.95	125.05	135.02	130.16	134.69
C4	121.06	125.35	121.48	125.73	129.30	138.58	122.46	127.50	129.49	138.11	126.39	137.86
C5	129.54	134.69	122.02	133.23	124.87	134.58	120.19	132.92	122.48	133.22	124.48	131.10
C6	114.47	113.72	113.71	116.12	116.34	114.68	112.16	113.65	114.96	116.06	112.76	113.59
C7	64.45	64.65	65.13	65.36	64.80	64.97	65.44	65.84	65.37	65.63	65.08	65.14
C8	170.33	175.44	169.95	174.74	170.11	175.04	169.60	174.27	169.65	174.43	170.23	175.40
H1	13.01	6.30	13.15	6.33	13.07	6.30	13.20	6.32	13.16	6.34	13.02	6.31
H2	6.92	7.23	-	-	6.94	7.15	-	-	-	-	-	-
H3	7.29	7.48	7.42	7.53	7.31	7.33	-	-	7.50	7.41	7.18	7.27
H4	6.96	7.17	6.95	7.10	-	-	7.19	7.14	-	-	-	-
H5	7.29	7.53	7.25	7.42	7.31	7.37	7.26	7.30	7.29	7.29	7.14	7.20
H6	6.92	6.86	7.01	6.87	6.94	6.75	7.02	6.73	7.04	6.74	6.82	6.63
H7	4.67	4.59	4.81	4.62	4.69	4.54	4.86	4.57	4.82	4.59	4.70	4.56
H8	4.67	4.59	4.81	4.62	4.69	4.54	4.86	4.57	4.82	4.59	4.70	4.56

**Table 6:** Minimum inhibitory (MIC) and minimum bactericidal (MBC) concentrations of herbicides and antibiotics studied against two certified soil bacterial strains obtained by the microdilution method.

Bacterial Strains	*Bacillus megaterium* ATCC 14581		*Pseudomonas aeruginosa* ATCC 15442	
	MIC	MBC	MIC	MBC
Herbicides	mg/mL			
PA	1.56	1.56	3.13	3.13
2CPA	1.56	1.56	3.13	3.13
4CPA	0.78	BS	3.13	3.13
2,3D	0.78	1.56	6.25	6.25
2,4D	0.78	1.56	3.13	3.13
MCPA	0.39	BS	3.13	3.13
Gentamycin	<1.91·10^−6^	BS	1.95·10^−3^	NT
Rifampicin	<1.91·10^−6^	BS	1.60·10^−2^	NT

BS—bacteriostatic mode of action; NT—not tested.

## Data Availability

The data presented in this study are available upon request from the corresponding author.

## Supplementary Materials

The following supporting information can be downloaded at: https://www.mdpi.com/article/10.3390/ma18071680/s1, Table S1: Electronic charge distribution (NBO and ChelpG) calculated of B3LYP/6-311++G(d,p) method in vaccum and wather solution (model CPCM) for phenoxyacetic acid and chlorinated derivatives, Figure S1 A: 1HNMR and 13CNMR for phenoxyacetic acid, Figure S1 B: 1HNMR and 13CNMR for 2-chlorophenoxyacetic acid, Figure S1 C: 1HNMR and 13CNMR for 4-chlorophenoxyacetic acid, Figure S1 D: 1HNMR and 13CNMR for 2,3-dichlorophenoxyacetic acid, Figure S1 E: 1HNMR and 13CNMR for 2,4-dichlorophenoxyacetic acid, Figure S1 F: 1HNMR and 13CNMR for 4-metyl-2-chlorophenoxyacetic acid, Figure S2: Normal modes for benzene ring calculated in B3LYP/6-311++G(d,p).
