# Supplementary material for: Study of the Relationship Between the Structures and Biological Activity of Herbicides Derived from Phenoxyacetic Acid
Source: Materials (Basel). 2025 Apr 7;18(7):1680. doi: 10.3390/ma18071680 (PMC11990099; doi:10.3390/ma18071680)
Supplement: Supplementary file 1 [file materials-18-01680-s001.zip › materials-3543252-supplementary.pdf]

Study of the relationship between the structures and biological activity of herbicides derived from phenoxyacetic acid

Grzegorz Świderski [1]*, Natalia Kowalczyk [1], Gabriela Tyniecka [1], Monika Kalinowska [1], Renata Łyszczek [2], Aleksandra Bocian [3], Ewa Ciszkowicz [3], Leszek Siergiejczyk [4], Małgorzata Pawłowska [5], Jacek Czerwiński [5]

Supplementary

Table S1. Electronic charge distribution (NBO and ChelpG) calculated of B3LYP/6-311++G(d,p) method in vaccum and wather solution (model CPCM) for phenoxyacetic acid and chlorinated derivatives

**Atom**	**PA**		**2CPA**		**4CPA**		**2,3D**		**2,4D**		**MCPA**	
**No.**	**NBO**	**CHelpG**	**NBO**	**CHelpG**	**NBO**	**CHelpG**	**NBO**	**CHelpG**	**NBO**	**CHelpG**	**NBO**	**CHelpG**
**C1**	0.318	0.516	0.297	0.506	0.317	0.502	0.316	0.507	0.299	0.505	0.319	0.298
	**0.315**	**0.511**	**0.295**	**0.524**	**0.317**	**0.490**	**0.318**	**0.511**	**0.298**	**0.506**	**0.319**	**0.305**
**C2**	-0.235	-0.310	-0.075	-0.097	-0.225	-0.308	-0.110	-0.089	-0.068	-0.093	-0.043	0.048
	**-0.294**	**-0.309**	**-0.081**	**-0.099**	**-0.226**	**-0.294**	**-0.114**	**-0.094**	**-0.064**	**-0.096**	**-0.043**	**0.053**
**C3**	-0.184	-0.015	-0.205	0.002	-0.211	-0.016	-0.052	0.140	-0.232	-0.022	-0.217	-0.180
	**-0.192**	**-0.016**	**-0.212**	**-0.017**	**-0.212**	**-0.034**	**-0.055**	**0.140**	**-0.233**	**-0.018**	**-0.213**	**-0.185**
**C4**	-0.232	-0.190	-0.217	-0.204	-0.069	0.006	-0.236	-0.183	-0.055	0.000	-0.060	0.065
	**-0.241**	**-0.190**	**-0.221**	**-0.209**	**-0.056**	**0.024**	**-0.240**	**-0.182**	**-0.052**	**-0.001**	**-0.056**	**0.073**
**C5**	-0.182	-0.049	-0.186	-0.017	-0.207	-0.018	-0.173	-0.046	-0.205	-0.021	-0.219	-0.095
	**-0.187**	**-0.055**	**-0.186**	**-0.036**	**-0.207**	**-0.033**	**-0.167**	**-0.046**	**-0.206**	**-0.021**	**-0.220**	**-0.099**
**C6**	-0.293	-0.322	-0.277	-0.353	-0.271	-0.330	-0.283	-0.337	-0.252	-0.347	-0.266	-0.259
	**-0.293**	**-0.314**	**-0.272**	**-0.342**	**-0.270**	**-0.318**	**-0.273**	**-0.338**	**-0.251**	**-0.345**	**-0.265**	**-0.263**
**C7**	-0.127	0.280	-0.128	0.313	-0.135	0.275	-0.128	0.313	-0.136	0.285	-0.134	0.190
	**-0.133**	**0.266**	**-0.134**	**0.298**	**-0.134**	**0.268**	**-0.135**	**0.298**	**-0.135**	**0.270**	**-0.133**	**0.184**
**C8**	-0.787	0.801	0.786	0.717	0.807	0.792	0.786	0.772	0.807	0.784	0.807	0.791
	**0.806**	**0.809**	**0.805**	**0.789**	**0.806**	**0.801**	**0.805**	**0.781**	**0.805**	**0.796**	**0.805**	**0.801**
**O1**	-0.562	-0.617	-0.555	-0.530	-0.607	-0.610	-0.554	-0.599	-0.604	-0.605	-0.607	-0.609
	**-0.616**	**-0.627**	**-0.613**	**-0.618**	**-0.614**	**-0.621**	**-0.611**	**-0.611**	**-0.611**	**-0.617**	**-0.615**	**-0.621**
**O2**	-0.697	-0.699	-0.697	-0.678	-0.693	-0.698	-0.696	-0.696	-0.692	-0.697	-0.693	-0.589
	**-0.688**	**-0.697**	**-0.678**	**-0.696**	**-0.687**	**-0.697**	**-0.686**	**-0.693**	**-0.686**	**-0.694**	**-0.688**	**-0.686**
**O3**	-0.532	-0.557	-0.519	-0.484	-0.543	-0.544	-0.517	-0.547	-0.532	-0.532	-0.543	-0.425
	**-0.549**	**-0.555**	**-0.536**	**-0.543**	**-0.544**	**-0.543**	**-0.532**	**-0.543**	**-0.532**	**-0.528**	**-0.544**	**-0.427**
**H1**	0.487	0.480	0.488	0.451	0.502	0.582	0.489	0.485	0.503	0.485	0.502	0.479
	**0.505**	**0.483**	**0.506**	**0.489**	**0.505**	**0.486**	**0.506**	**0.489**	**0.505**	**0.489**	**0.505**	**0.483**
**H2**	0.222	0.154	-	-	0.230	0.169	-	-	-	-	-	-
	**0.223**	**0.154**	**-**	**-**	**0.230**	**0.167**	**-**	**-**	**-**	**-**	**-**	**-**
**H3**	0.206	0.095	0.222	0.090	0.230	0.113	-	-	0.243	0.120	0.225	0.140
	**0.215**	**0.095**	**0.230**	**0.111**	**0.231**	**0.118**	**-**	**-**	**0.244**	**0.121**	**0.226**	**0.143**
**H4**	0.206	0.113	0.211	0.121	-	-	0.225	0.133	-	-	-	-
	**0.215**	**0.113**	**0.221**	**0.135**	**-**	**-**	**0.234**	**0.134**	**-**	**-**	**-**	**-**
**H5**	0.204	0.110	0.208	0.096	0.230	0.114	0.212	0.132	0.234	0.123	0.228	0.126
	**0.214**	**0.112**	**0.219**	**0.117**	**0.230**	**0.118**	**0.225**	**0.133**	**0.234**	**0.121**	**0.229**	**0.128**
**H6**	0.208	0.157	0.212	0.148	0.229	0.169	0.214	0.174	0.234	0.184	0.228	0.161
	**0.223**	**0.155**	**0.228**	**0.171**	**0.229**	**0.168**	**0.231**	**0.173**	**0.234**	**0.184**	**0.228**	**0.163**
**H7**	0.201	0.028	0.203	0.005	0.217	0.033	0.204	0.029	0.220	0.034	0.216	0.051
	**0.216**	**0.032**	**0.219**	**0.029**	**0.217**	**0.035**	**0.221**	**0.033**	**0.220**	**0.038**	**0.217**	**0.052**
**H8**	0.201	0.028	0.203	0.005	0.217	0.033	0.204	0.029	0.220	0.034	0.216	0.051
	**0.216**	**0.032**	**0.219**	**0.029**	**0.217**	**0.035**	**0.221**	**0.033**	**0.220**	**0.038**	**0.217**	**0.052**
**Cl2**	-		0.029	-0.089	-	-	0.064	-0.086	0.021	-0.099	-	-

	**-**	**-0.002**	**-0.128**	**-**	**-**	**0.034**	**-0.088**	**0.015**	**-0.103**	**-**	**-**
**Cl3**	-		-	-	-	0.035	-0.130	-	-	-	-
	**-**		**-**	**-**	**-**	**0.019**	**-0.131**	**-**	**-**	**-**	**-**
**Cl4**	-		-	-0.018	-0.164	-	-	-0.004	-0.140	-0.022	-0.170
	**-**		**-**	**-0.022**	**-0.170**	**-**	**-**	**-0.007**	**-0.142**	**-0.027**	**-0.173**

bold – values calculated for water model CPCM

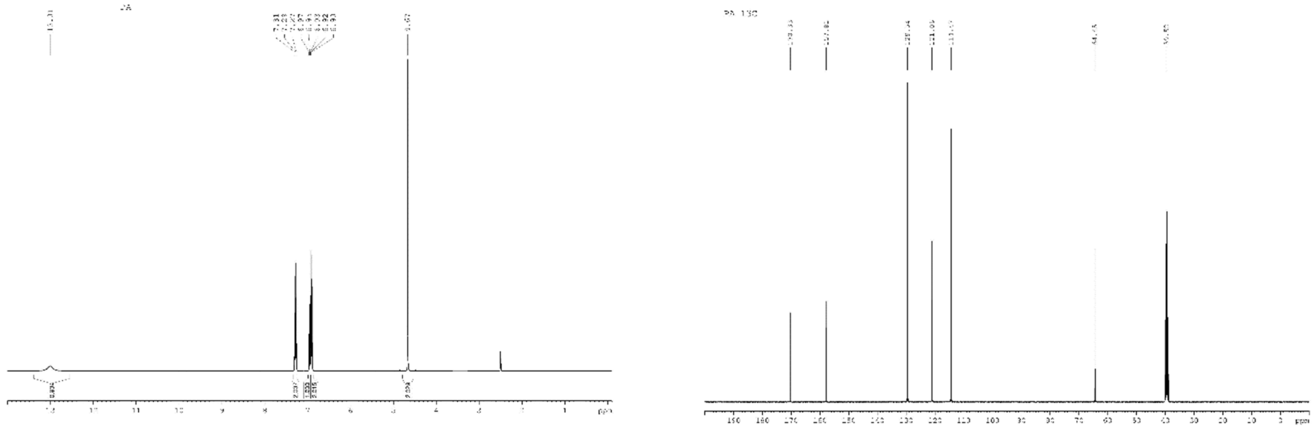

Figure S1 A. 1HNMR and 13CNMR for phenoxyacetic acid

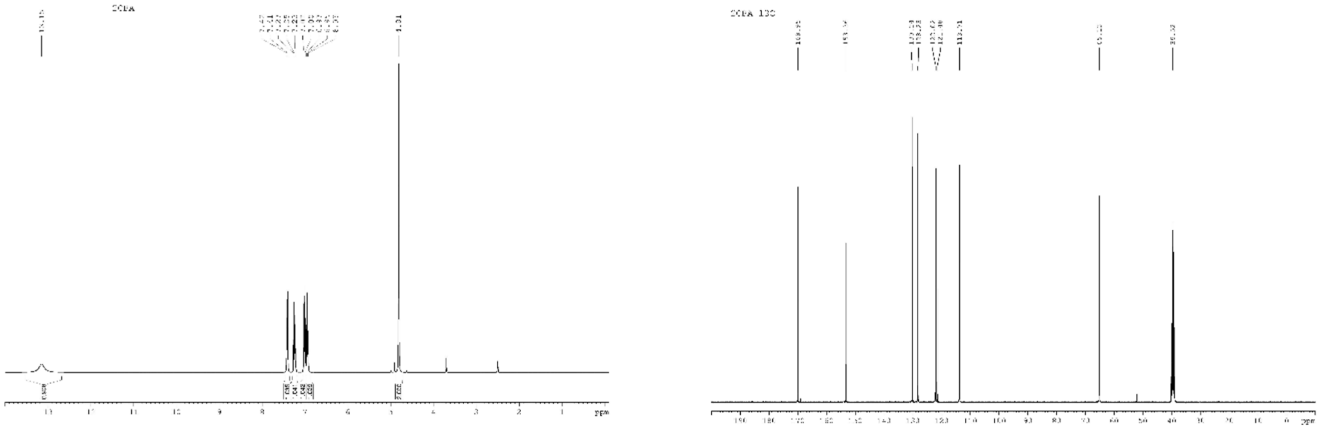

Figure S1 B. 1HNMR and 13CNMR for 2-chlorophenoxyacetic acid

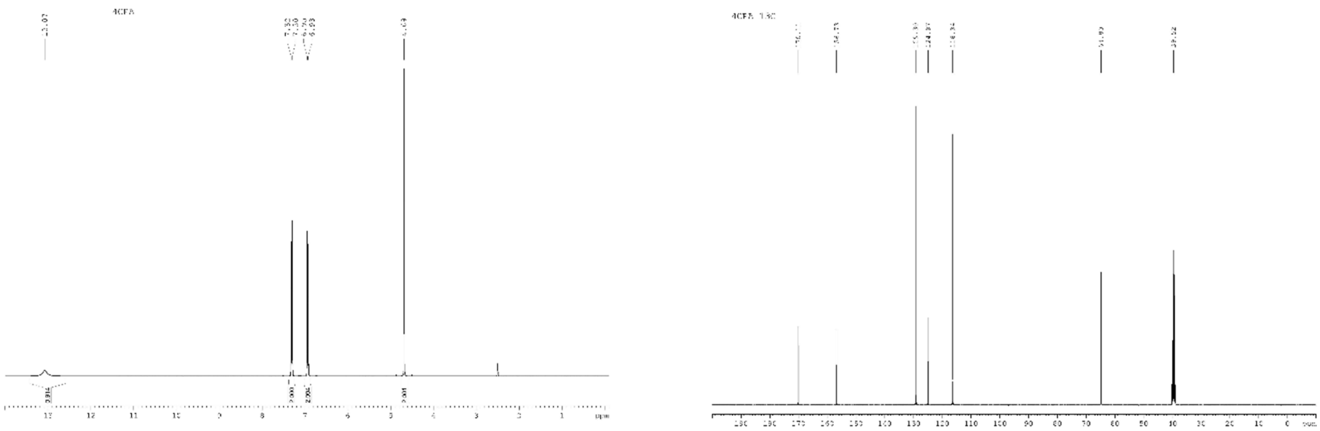

Figure S1 C. 1HNMR and 13CNMR for 4-chlorophenoxyacetic acid

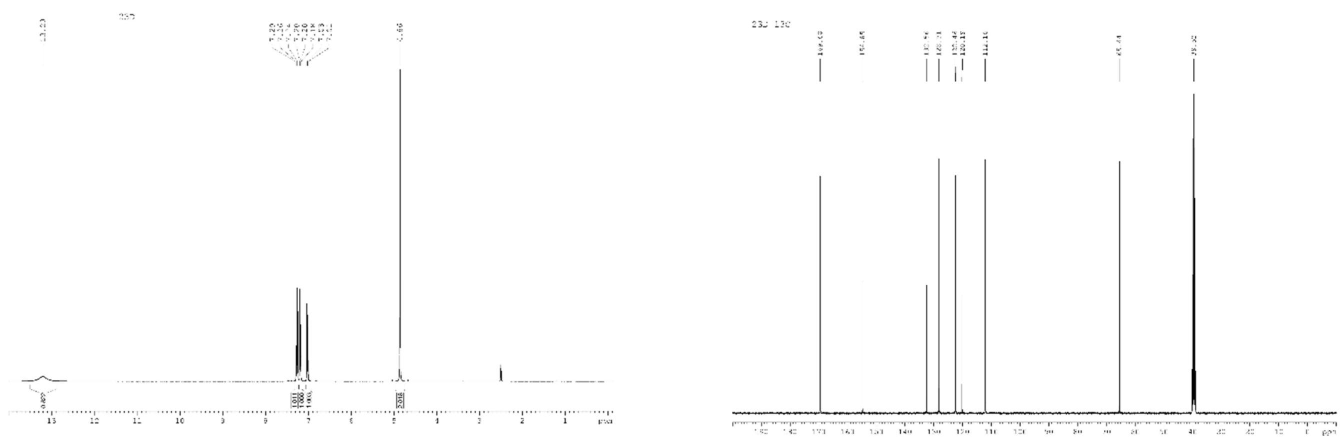

Figure S1 D. 1HNMR and 13CNMR for 2,3-dichlorophenoxyacetic acid

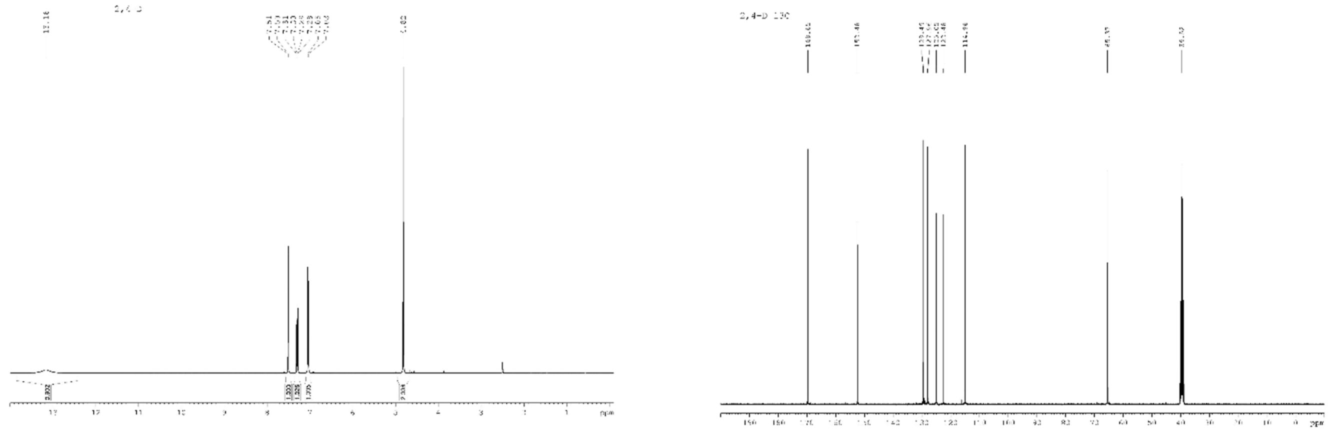

Figure S1 E. 1HNMR and 13CNMR for 2,4-dichlorophenoxyacetic acid

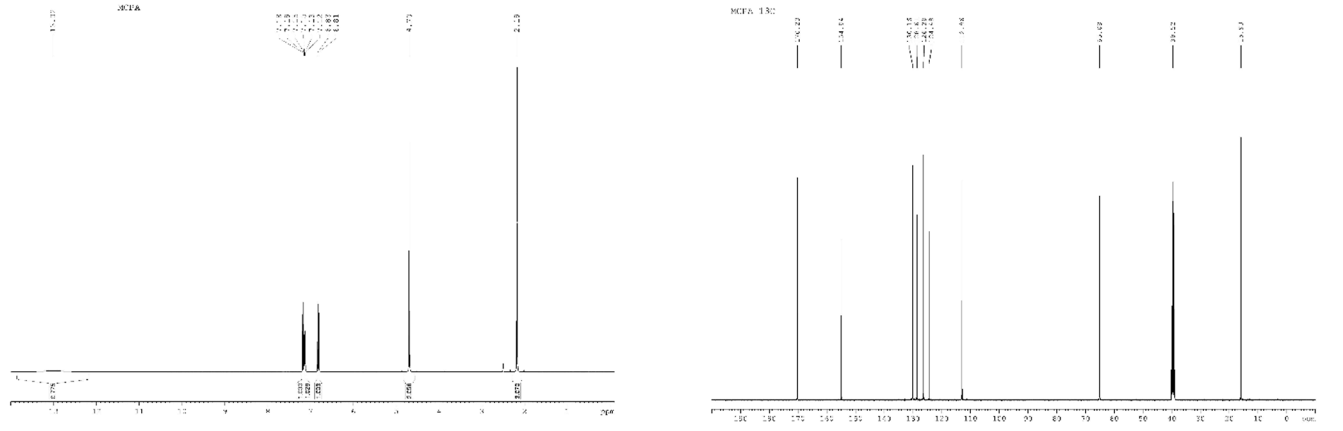

Figure S1 F. 1HNMR and 13CNMR for 4-metyl-2-chlorophenoxyacetic acid

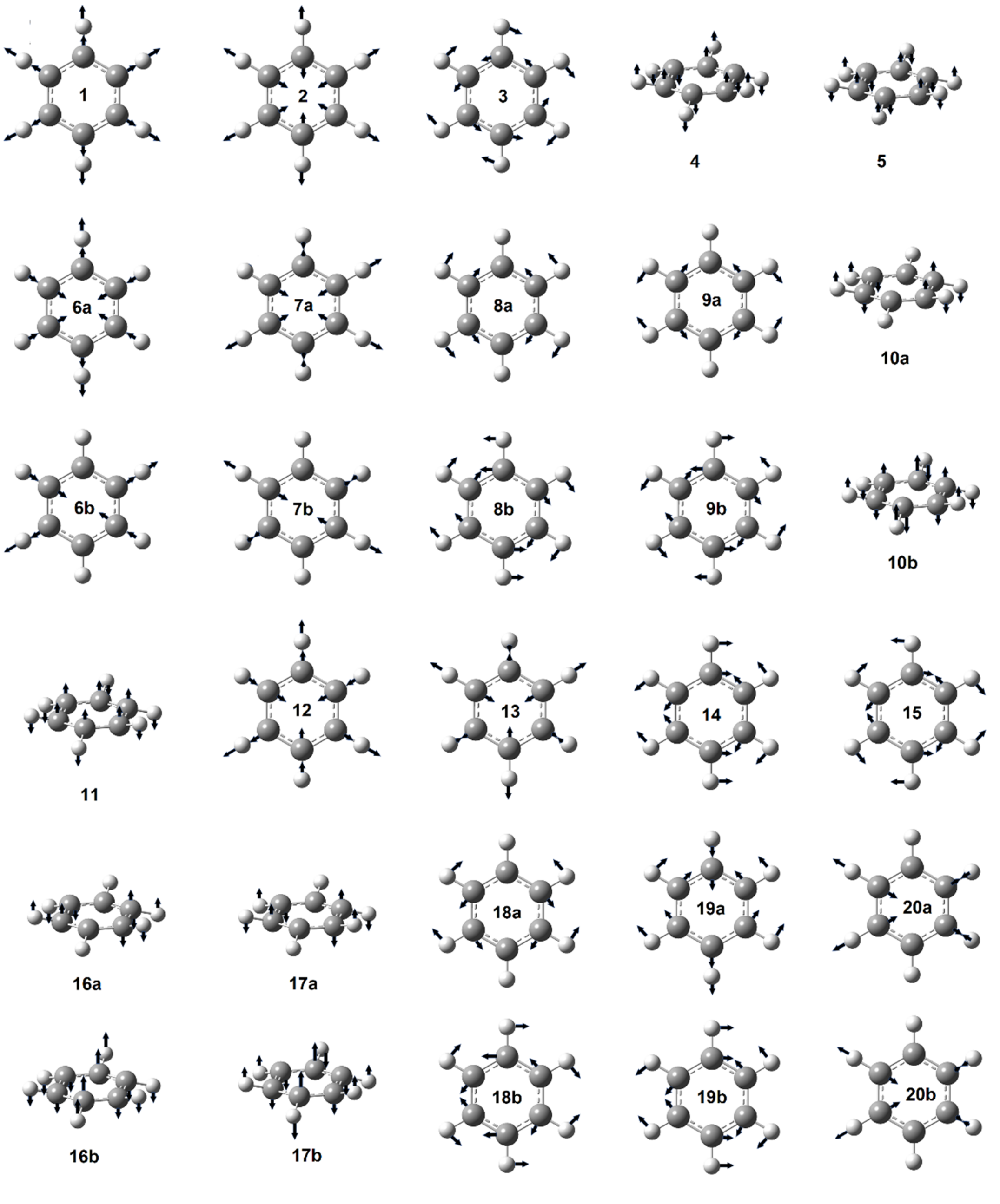

Figure S2. Normal modes for benzene ring calculated in B3LYP/6-311++G(d,p)
